# Apoptosis gene profiling reveals spatio-temporal regulated expression of the p53/Mdm2 pathway during lens development^☆^

**DOI:** 10.1016/j.exer.2009.01.020

**Published:** 2009-06-01

**Authors:** Jenny C. Geatrell, Peng Mui (Iryn) Gan, Fiona C. Mansergh, Lilian Kisiswa, Miguel Jarrin, Llinos A. Williams, Martin J. Evans, Mike E. Boulton, Michael A. Wride

**Affiliations:** a School of Optometry and Vision Sciences, Cardiff University, Maindy Road, Cardiff, Wales CF24 3LU, UK; b School of Biosciences, Cardiff University, Museum Avenue, Cardiff, Wales CF10 3US, UK; c Smurfit Institute of Genetics, Trinity College Dublin, Dublin 2, Ireland; d Mason Eye Institute, One Hospital Drive, Columbia, Columbia University, MO 65212, USA; e Department of Anatomy and Cell Biology, University of Florida, Gainesville, Florida, USA; f Department of Zoology, School of Natural Sciences, Trinity College Dublin, Dublin 2, Ireland

**Keywords:** mouse, chick, lens, development, apoptosis, array, p53, Mdm2, Huntingtin

## Abstract

Evidence is emerging for apoptosis gene expression in the lens during development. Therefore, here we used a filter array to assess expression of 243 apoptosis-related genes in the developing postnatal mouse lens using ^33^P labelled cDNA synthesized from p7 and p14 mouse lenses. We demonstrated that 161 apoptosis-related genes were expressed at levels significantly above background and 20 genes were potentially significantly differentially expressed (*P* < 0.05) by at least 2-fold between p7 and p14. We used RT-PCR to confirm expression of these genes in newborn, p7, p14 and 4 wk mouse lens cDNA samples. Expression of 19/20 of the genes examined was confirmed, while 5 genes (*Huntingtin*, *Mdm2*, *Dffa*, *galectin-3* and *Mcl-1)* were confirmed as differentially regulated between p7 and p14. RT-PCR was also used to examine the expression of the chick homologues of the most-highly expressed and/or potentially differentially regulated genes in chick embryo lenses at E6–E16. The majority of genes expressed in the postnatal mouse lens were also expressed in the chick embryo lens. Western blotting confirmed developmentally regulated expression of Axl and Mcl-1 during mouse lens development and of Mdm2, Mdm4/X and p53 during mouse and chick lens development. Western blotting also revealed the presence of p53 and Mdm4/X splice variants and/or proteolytic cleavage products in the developing lens. Since Mdm2 is a regulator of the tumour suppressor gene p53, we chose to thoroughly investigate the spatio-temporal expression patterns of p53, Mdm2 and the functionally related Mdm4/X in mouse lens development at E12.5–E16.5 using immunocytochemistry. We also examined Mdm2 expression patterns during chick lens development at E6-E16 and Mdm4/X and p53 at E14. Expression of Mdm2, Mdm4/X and p53 was spatio-temporally regulated in various compartments of the developing lens in both mouse and chick, including lens epithelial and lens fibre cells, indicating potential roles for these factors in regulation of lens epithelial cell proliferation and/or lens fibre cell differentiation This study provides a thorough initial analysis of apoptosis gene expression in the postnatal mouse lens and provides a resource for further investigation of the roles in lens development of the apoptosis genes identified. Furthermore, building on the array studies, we present the first spatio-temporal analysis of expression of p53 pathway molecules (p53, Mdm2 and Mdm4/X) in both developing mouse and chick lenses, suggesting a potential role for the p53/Mdm2 pathway in lens development, which merits further functional analysis.

## 1 Introduction

Lens development occurs throughout the lifetime of the individual and involves the terminal differentiation of lens epithelial cells into lens fibre cells (Piatigorsky, 1981; Wride, 1996). This process begins during embryogenesis and continues, albeit at a slower rate, into adulthood and old age. A number of characteristic morphological changes are observed in lens fibre cells during differentiation. The cells increase in length by 50- to 100-times, accompanied by an increase in fibre-specific proteins, including intermediate filament proteins CP49 and CP95 (Ireland et al., 2000) and crystallins (Cvekl and Piatigorsky, 1996).

The elimination of potentially light-scattering intracellular organelles, including nuclei and all associated nucleic acid, is a key feature of the differentiation of lens epithelial cells into fibre cells and is thought to involve at least some components of the apoptosis signalling pathway (Dahm, 1999; Wride et al., 1999, 2003; Wride, 2000, 2007; Bassnett, 2002, 2008). However, unlike in ‘conventional’ apoptosis, the cells from which the organelles have been removed persist throughout life, rather than being destroyed. Additional structural differences have been observed, including the persistence of the cytoskeleton in mature fibre cells, whereas it is completely degraded during apoptosis (Bassnett and Beebe, 1992; Dahm et al., 1998). Also, there is no flipping of phosphatidylserine to the outer membrane of the lens fibre cells as observed in apoptosis (Bassnett and Mataic, 1997; Wride and Sanders, 1998). Finally, in executioner caspase (caspase-3, -6 and -7) knockout mice, lens fibre cell organelle loss proceeds as normal (Zandy et al., 2005).

Cataract occurs when opacities form in the normally transparent lens and is the commonest cause of blindness worldwide (Francis et al., 1999, 2000; Congdon, 2001). Lens opacities can be congenital or appear during ageing and can form as a result of genetic mutations or exposure to toxic insults; e.g. UV radiation. Many of the genetic mutations causing cataract affect structural and/or transparency related components of the lens (e.g. connexins and crystallins, Graw and Loster, 2003). Congenital cataracts are rare in developed countries (30 cases per 100,000 births) (Graw, 2004). Moreover, maternal rubella virus infection causes bilateral congenital cataract (Gregg and Banatvala, 2001; McAlister Gregg, 2001), possibly as a result of defects in lens fibre cell organelle degradation. Accumulation of nuclear and mitochondrial fragments in cortical cataract can occur due to incomplete organelle degradation in the equatorial region of the lens (Pendergrass et al., 2005, 2006). Prevention of DNA degradation in a mouse model, due to DNase II-like acid DNase (DLAD) deficiency leads to DNA accumulation in the lens, thereby causing cataract (Nishimoto et al., 2003). Therefore, DLAD must be the DNase responsible for nuclear degradation during lens cell differentiation (Nishimoto et al., 2003; Nakahara et al., 2007).

Microarray studies have been used to profile gene expression in the lens during early postnatal development in order to compare gene expression therein with non-lens tissues and to compare gene expression profiles in lens compartments at different stages of maturation (Wride et al., 2003; Ivanov et al., 2005; Xiao et al., 2006). This technology has also pinpointed gene expression changes between cataractous and normal age-matched lenses in humans and in mouse models of cataract, including the Sparc and Mimecan knockouts (Hawse et al., 2003; Ruotolo et al., 2003; Hawse et al., 2004; Mansergh et al., 2004; Segev et al., 2004). These studies demonstrated significant differential gene expression between cataractous lenses and age-matched controls. Expression of many unexpected genes has been identified in the lens using arrays, including those encoding the haemoglobin subunits (Wride et al., 2003; Mansergh et al., 2004, 2008). Notably, study of genes expressed in normal lens development highlighted the presence of many genes associated with apoptotic processes.

Here, we have used nylon arrays comprised of 243 cDNAs representing genes with known roles in apoptosis in order to carry out an initial screen of the expression of these genes at postnatal day 7 (p7) and postnatal day 14 (p14) of mouse lens development. These stages were chosen as the postnatal period before day 14 is a period of rapid lens growth, accompanied by lens fibre cell differentiation and organelle loss involving apoptosis signalling pathways (Wride, 2000). Formation of the organelle free zone (OFZ) is complete at p14 when the eyes open, allowing for clear vision (Kuwabara and Imaizumi, 1974). A number of highly expressed or differentially regulated genes were selected for follow-up using RT-PCR. In order to further select for biological relevance via cross-species comparison, we tested expression of the chick homologues of selected genes during chick embryo lens development (E6–E16) using RT-PCR.

The *mouse double minute 2* (*Mdm 2*) gene, the product of which is a regulator of p53, was differentially regulated between the two stages studied in the mouse lens, while *p53* itself was also highly expressed. Mdm4/X, was not printed on the array used, but is known to be intimately functionally related to both p53 and Mdm2 (Marine et al., 2006). The p53 pathway is a key component of apoptotic signalling; p53 is possibly the most pivotal tumour suppressor gene and its ablation is a primary cause of cancer (Toledo and Wahl, 2007). Furthermore, it is becoming apparent that p53/Mdm2 signalling is involved in various developmental processes including osteoblast differentiation (Lengner et al., 2006), nervous system development (Xiong et al., 2006) and in regulating proliferation and progenitor expansion in various cell lineages (Liu et al., 2007). The role of p53 family molecules in embryonic development has recently been reviewed (Danilova et al., 2008a,b) and it was suggested that a significant number of congenital developmental abnormalities may be due to defects in the p53 protein family. Furthermore, there is some evidence that the p53 pathway may be involved in eye and/or lens development. P53 expression has been demonstrated in the normal adult mouse eye in the corneal epithelium (Tendler et al., 2006) and in the lens epithelial cells of the central and pre-equatorial zones and in the lens fibre nuclear bow region (Pokroy et al., 2002), while increased p53 expression in the rat lens epithelium following exposure to UV light has been associated with apoptosis and cataract (Ayala et al., 2007). Furthermore, temporally distinct patterns of p53-dependent apoptosis have been identified during mouse lens development (Pan and Griep, 1995) and overexpression of human wild-type p53 in the mouse lens results in defects in lens fibre cell differentiation (Nakamura et al., 1995). However, there is no prior evidence for Mdm2 expression in the lens and the spatio-temporal pattern of expression of members of the p53 pathway in lens development remains undetermined. In the latter half of the studies presented here, we therefore focused on Mdm2, p53 and Mdm4/X in Western blotting and immunocytochemistry studies during mouse and chick lens development.

## 2 Materials and methods

### 2.1 Collection of lenses

Mice (129SvEv) were maintained on a 12 h light/12 h dark light cycle with food and water *ad libitum* and were handled according to Home Office UK guidelines. Lenses were extracted from mice at different stages of maturation: newborn (Nb), postnatal day 7 (P7), postnatal day 14 (P14) and 4 wk (4wk). Mice were cervically dislocated and enucleated. The lenses were then removed from a posterior incision in the eyeballs under a research stereo microscope (Nikon SMZ800) using No. 5 forceps (Sigma, UK) and pooled for RNA sample collection. A different litter was used for each pooled lens RNA sample collected.

Lenses were also collected from White Leghorn chick embryos (Henry Stewart and Co., Lincolnshire, UK). Fertile eggs were placed in a humidity-controlled incubator (Brinsea Octagon 100, Jencons, UK) at 37.8 °C. Embryos were placed at −4 °C for 20–30 min to cool, then decapitated using a fresh scalpel blade before the lenses were removed using tungsten needles under a dissecting microscope (Nikon SMZ800). Lenses were collected from both eyes at embryonic days (E) 6, 8, 10, 12, 14 and 16 and were pooled to generate each RNA sample.

### 2.2 RNA extraction, quantification and integrity

Lenses were immediately homogenised in TRIzol^®^ reagent (Invitrogen, UK) using a tissue grinder (Wheaton) and RNA was isolated using the manufacturer's protocol. RNAs were quantified using a spectrophotometer (GeneQuant II, Pharmacia Biotech) at 260 nm and checked for RNA integrity via agarose gel electrophoresis by assessing 18 and 28S band intensities.

### 2.3 Apoptosis arrays: experimental design and MIAME standards

We used Panorama™ mouse apoptosis arrays (Sigma-Genosys, UK; cat# G1039), in conjunction with RNA extracts from p7 and p14 mice. These arrays consisted of nylon membranes on which cDNAs representing 243 known apoptosis-related genes were printed. Four biological repeats were carried out for day 7, and 3 for day 14 lenses. Each array carried duplicate spots, giving 8 and 6 repeats respectively. These arrays comply with MIAME standards (Brazma et al., 2001); the array platform and all data described in this paper were submitted to the GEO database http://www.ncbi.nlm.nih.gov; GEO accession: GSE8731. The complete list of genes present on the array is provided in Supplementary Table 1.

### 2.4 Array hybridisation

Radiolabelled cDsNAs were synthesized from the purified RNA according to the manufacturer's instructions (Sigma-Genosys, UK) incorporating ^33^P-dCTP (Amersham Biosciences, UK). Arrays were first pre-hybridised to prevent non-specific binding of DNA by washing in 50 ml 2 × SSPE (Sigma, UK) at room temperature for 5 min then in hybridisation solution (Sigma, UK) containing salmon testes DNA (100 μl Salmon testes DNA in 10 ml hybridisation solution) at 65 °C for at least an hour before the addition of the radiolabelled cDNA.

Unincorporated radiolabelled nucleotides were removed using a sephadex bead containing spin column (Sigma-Genosys, UK) and centrifugation.

Purified radiolabelled cDNA was then added to 2–3 ml hybridisation solution (5 × SSPE, 2% SDS, 5 × Denhardt's reagent, 100 μg/ml sonicated denatured salmon testes DNA) and denatured by heating at 95 °C for 10 min and then added to the arrays, which were hybridised overnight for 18 h in a hybridisation oven (UVP, HC-3000 Hybricycler) at 65 °C. The hybridisation solution was decanted and arrays were washed with solution I (0.5 × SSPE; 1% SDS), 3 × 2–3 min each. Wash solution I was then used to wash the arrays at 65 °C, 2 × 20 min. Arrays were then washed for a further 20 min at 65 °C using solution II (0.1 × SSPE, 1% SDS). The wash solution was discarded and the arrays were wrapped in clingfilm before placing into a storage phosphor screen (Amersham Biosciences, UK) for 5–7 days. The phosphor screen was scanned using a Typhoon scanner (Amersham Biosciences, Typhoon 9410 Variable Mode Imager); a phosphoimage of an array hybridised with P7 radiolabelled RNA is presented in Supplementary Fig. 1.

Some arrays were subsequently stripped using boiling stripping solution (10 mM Tris–HCl, 1 mM EDTA, 1% SDS, pH 8; Sigma, UK). Stripped arrays were wrapped in clingfilm and exposed to the phosphor screen in order to check that all radiolabelled cDNA had been removed before re-use. Scatter plots providing an overview of the reproducibility of array results between repetitions are presented in Supplementary Fig. 2.

### 2.5 Array analysis

Array images were analyzed using ImaGene 5 (Biodiscovery) and spot intensity and a background signal values for each individual spot were determined. The background value for each spot was calculated as a mean of the intensity of a set number of pixels surrounding the spot. The individual background values were then subtracted from the corresponding spot intensity, to give a corrected intensity value (i.e. corrected intensity = original spot intensity minus background value for that spot). Data were subsequently exported to Microsoft^®^ Excel for further analysis. The mean and standard deviation (+2SD) spot intensity values were calculated for each spot; these values were subsequently used to filter the data. Spots with signals lower than mean + 2SD of the background were removed from the data set following normalisation. Each corrected spot value was normalised before being filtered so that the spot intensities could be compared between arrays. Two different approaches for normalisation were used: 1) housekeeping gene normalisation, 2) global normalisation. In the first approach, the spots were normalised with respect to the mean value for the housekeeping genes on each array. In global normalisation, the spots were normalised using the mean spot intensity calculated for all spots on each array excluding housekeeping genes, negative controls and positive controls.

Following normalisation, a mean intensity value was calculated for each gene on each array at each time point. The mean values were compared between P7 and P14 using an unpaired Student's *t*-test. Genes were considered to be significantly differentially regulated if they showed a 2-fold or greater difference between the time points and *p* < 0.05. Using the housekeeping gene normalisation method, 20 genes were significantly differentially regulated, while with global normalisation 60 genes were significantly differentially regulated. The latter data set also included the 20 genes identified using the housekeeping gene method. Therefore, since the housekeeping gene method of normalisation was more stringent, this was the preferred method. In order to estimate which genes were highly expressed at each stage, we asked that at least one of the spots representing each gene be above background + 2SD in all replicates for a given stage (p7 or p14). Analysis of all p7 repeats versus all p14 repeats was carried out by *t*-test, we also required a fold change of 2. Statistically significant, differentially regulated genes were tested by PCR using 3 biological replicates for each stage.

### 2.6 RT-PCR confirmations

Before cDNA synthesis, DNase digestion was completed using the TURBO DNase protocol (Ambion, UK) according to the manufacturer's instructions. cDNA was generated using the Superscript™ First-Strand Synthesis System (Invitrogen), also according to the manufacturer's instructions. Mouse PCR primers were designed using Primer3 (http://frodo.wi.mit.edu/cgi-bin/primer3/primer3_www.cgi) from Genbank reference sequence for each gene. The mouse housekeeping gene (Gapdh) used for the PCR confirmations was taken from Mansergh et al. (2004). Primers were obtained from Operon and were resuspended in nuclease free water (Sigma, UK). Chick primers were also designed as above. However, for sequences unavailable on Gene, BLAST searches were used to identify the likely chick homologues. The primer sequences for the housekeeping gene used, Gapdh, was taken from (Faulkner-Jones et al., 2003). The mouse and chick primer sequences are shown in Tables 1 and 2 respectively.

The GoTaq^®^ protocol (Promega, UK) was used for PCRs using standard procedures. Equal loading of cDNA was monitored using the products of the gapdh PCR reaction and subsequent image analysis. Adjustments were made to the amount of cDNA used in the PCR reactions until the resulting bands from the gapdh PCR reaction were shown to be of the same intensity in each cDNA sample used at a minimum number of cycles. The semi-quantitative RT-PCR method used was similar to that described in our previous publications (Wride et al., 2003; Mansergh et al., 2004). For each gene, the number of cycles used for each set of primers was based on initial experiments in which the number of PCR cycles was varied such that, for all genes, the PCRs were in the linear part of the PCR amplification curve (examples of this for 3 genes are shown in Supplementary Fig. 3). We have recently demonstrated that the semi-quantitative method we describe here is robust, since we have revealed similar differential patterns of expression of the chick *Survivin* gene during chick lens development using both semi-quantitative RT-PCR and QPCR (Jarrin et al., unpublished data).

### 2.7 PAGE/Western blotting

Protein was isolated from pooled postnatal mouse lenses or embryonic chick lenses using RIPA buffer (Upstate, USA) containing protease inhibitor cocktail (Sigma, UK). Samples were incubated at 4 °C on a rotator for 30 min and then centrifuged at 13,000×g for 30 min at 4 °C. The supernatant was removed, aliquoted and stored at −20 °C. Protein samples were quantified using a BCA assay (Pierce, UK) according to the manufacturer's protocol. SDS-PAGE was carried out using 10–15% gels using the Bio-Rad Mini-Protean^®^ 3 cell system. 10μg of protein sample was added to each well; a molecular weight marker was also loaded (Bio-Rad Precision Plus). Proteins were subsequently transferred to nitrocellulose membrane (Hybond™-ECL™, Amersham Biosciences, UK) using transfer conditions of 100 V, 350 mA for 45 min. Proteins were visualised on the membrane using Ponceau S (Sigma, UK) and white light photography (UVP BioDoc-It™ System). Membranes were then washed with 1 × TBS/Tween to remove the Ponceau S before the blocking (using 5% milk), washing and antibody incubation. The following rabbit polyclonal primary antibodies were all purchased from Santa Cruz Biotech and were used here in Westerns for 2 h each at a dilution of 1:200: Mcl-1 (sc-819), axl (sc-20741), MdmX (sc-28222), and p53 (sc-6243). The Mdm2 antibody (ab38618) used in Westerns was obtained from AbCam (Cambridge, UK) and was also used at 1:200. Goat anti-rabbit IgG (Santa Cruz, sc-2004; 1:5000) was used as a secondary antibody for each antibody above for 1 h. A goat polyclonal actin antibody (Santa Cruz, sc-1616; 1:5000) was also used with a donkey-anti goat IgG (Santa Cruz, sc-2020; 1:5000) as the secondary antibody. Antibodies were diluted in 1% skimmed milk. Bands were detected on film (Hyperfilm™, Amersham Biosciences, UK) using ECL Plus Western blotting detection reagents (Amersham Biosciences, UK). A number of different film exposure times ranging from 1 to 10 min were used. Membranes were stripped using standard procedures and reprobed using the actin antibody.

Autorads were scanned and images of Western blots saved as .tif files. Images were imported to Scion Image (Scion Corporation) for analysis of band intensities (measured as mean pixel intensity). Measurements from Scion Image were imported into Microsoft^®^ Excel in which intensities were normalised by dividing by the mean reading for all bands measured from a given sample set. Bands of an above average intensity are therefore above 1 while those below average are below 1 in value. This calculation also puts experimental readings and readings from beta-actin hybridised Westerns from the same samples on the same scale. Normalised sample readings were then divided by the value for beta-actin intensity from the same sample. Finally, means and standard deviations were calculated for all repetitions (at least *n* = 3 for each protein examined). Mean values, thus obtained, were then graphed using Microsoft^®^ Excel; error bars represent plus and minus half a standard deviation. The bar charts generated are provided as Supplementary Figs. 4, 6 and 7.

### 2.8 Tissue processing for immunocytochemistry

Chicken embryos (6 and 8 days, heads; 10–16 days, eyes) were incubated to the appropriate stage, removed from the eggs and placed in ice cold phosphate-buffered saline (PBS). Mouse embryos were collected at E12.5, E14.5 and E16.5 post-coitum. Mouse embryos were embedded whole. All tissues were washed with PBS. Either whole chick embryo heads or eyes were fixed and embedded depending on the stage. Eyes were removed from the embryo, cut centrally with a razor blade and the posterior segments of the eyes were discarded. Tissues were fixed for 24 h at 4 °C in 4% paraformaldehyde (PFA), then washed 2 × 30 min in PBS, dehydrated through a graded series of ethanol and cleared in 50:50 ethanol:xylene, 30 min and then 100% xylene, 3 min. Tissues were then infiltrated with paraffin wax and embedded in plastic moulds using standard procedures. Tissues were subsequently sectioned at 7–8 μm on a microtome (HM 325, Microm) and mounted on microscope slides (Fisher, UK).

### 2.9 Immunocytochemistry

Mouse and chick slides were dewaxed in xylene, then re-hydrated through a graded series of alcohol and washed 2 × 10 min each in PBS. Antigen retrieval was then carried out using a citric acid based antigen unmasking solution (Vector labs, UK) for 15 min in a pressure cooker. The sections were allowed to cool, then endogenous peroxidase activity was quenched with 5 ml Methanol 98%, 5 ml Hydrogen Peroxide 30% and 40 ml ddH2O, 5 min. Immunocytochemistry was carried out using the VECTASTAIN^®^ Elite Universal ABC kit (Vector labs, UK) according to the manufacturer's protocol. Primary rabbit polyclonal antibodies Mdm2 (H-221: sc-7918; Santa Cruz Biotech), MdmX (H-130: sc-28222; Santa Cruz Biotech), and p53 (FL-393: sc-6243; Santa Cruz Biotech) were incubated at a dilution of 1:50 in 1 × PBS, 4 h at room temperature. The sections were then stained for 4 min with Very intensive Purple (VIP; Vector labs, UK). Slides were dehydrated in a graded series of alcohol, cleared in xylene and then mounted in mounting medium (DPX, Raymond Lamb Laboratories, UK). Slides were coverslipped, allowed to dry and examined under bright field using a Leica DMRA2 microscope with attached digital camera.

## 3 Results

### 3.1 Array analysis: highly expressed genes

**Fig. 1:**
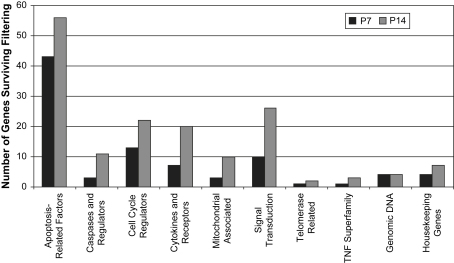
Gene families expressed in p7 and/or p14 mouse lenses at above-background levels as determined using Panorama™ apoptosis arrays before normalisation was applied. Genes have been grouped into gene families by Gene Ontology (Sigma-Genosys, UK). The mean background and standard deviation values were calculated for each spot representing a gene on the array. Spots were considered to be below background if their original intensity was lower than the mean background, plus 2 standard deviations of background and were therefore removed from the data set. For a complete list of genes at p7 and p14 surviving the filtering procedure, please see Supplementary Tables 2 and 3.

We identified 161 apoptosis-related genes using the arrays, which fell into several different gene ontology categories as defined by the manufacturer of the arrays (Fig. 1). The top 10 most-highly expressed genes at p7 and p14 are presented in Table 3.

The most abundant genes were those in the ‘apoptosis-related factors’ category (p7, 46 genes; p14, 56), including, amongst the most-highly expressed, *clusterin*, *Gpx1, Pin, Sarp-2/sfrp-1, Dad-1* and *Mts-1* (Table 3). There were several ‘caspases and regulators’ expressed (p7, 3; p14, 11), including *caspase-2*, *-3*, *-7* and *-8* as well as inhibitors of apoptosis (IAPs), such as *Survivin* and *Xiap* a genes encoding caspase substrates, *Parp* and *Parp-2*. We have previously shown by RT-PCR that Survivin is expressed in the lens and down-regulated during cataract progression (Mansergh et al., 2004).

There were also genes categorised as ‘cell cycle regulators’ that are also involved in apoptosis (p7, 13; p14, 22), such as *cyclin-G1* (expressed at very high levels; Table 3) as well as *Mdm2, p53* and the gene encoding p53-binding protein 2 (53Bp2). ‘Mitochondrial associated’ genes were identified (p7, 3; p14, 10) including *cytochome c* and the *Bcl-2* family members, *bax*, *Bag-1*, *Bak*, *Bcl-2* and *Bcl-w*. There were also significant numbers of ‘cytokines and receptors’ (p7, 7; p14, 20), including *Axl* and *TGF-β*, and genes involved in ‘signal transduction’ (p7, 10; p14, 26), including *Akt, Cradd*, *Fadd*, and *GSK3B*. Two ‘telomerase related’ genes were also identified (p7, 1; p14, 2), namely *TP1/Tep1* and *TR/TeRc* and three members of the ‘TNF superfamily’ (p7, 1; p14, 3), *NGFR*, *FasL* and *Tall1/Thank/Baff/TNFSF13B*.

The complete set of expressed genes at p7 and p14 is presented in Supplementary Tables 2 and 3 and the data are also available through the Gene Expression Omnibus (GEO) database: http://www.ncbi.nlm.nih.gov, GEO accession: GSE8731.

### 3.2 Array analysis: differentially expressed genes

**Fig. 2:**
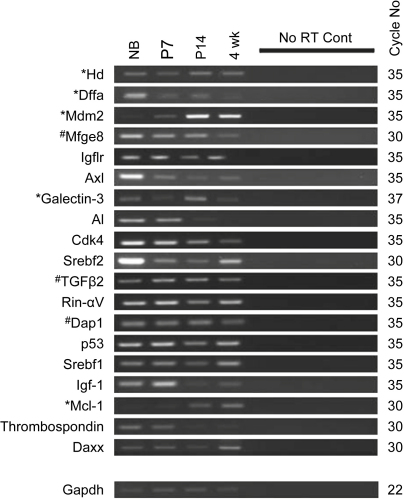
Semi-quantitative PCR results for differentially expressed genes identified using the arrays. Results are arranged in order (top to bottom) of the fold differences observed from the array results. Those genes (*Hd*, *Mdm2*, *Dffa*, *galectin-3* and *Mcl-1*) confirmed as up-regulated between p7 and p14 are labeled with an asterisk. Genes labeled with # indicate those that, although up-regulated between p7 and p14 according to the array, did not change in expression by RT-PCR. The remaining (un-labelled) genes, although up-regulated between p7 and p14 according to the array, were actually down-regulated between these two stages by RT-PCR. Results are representative of three repetitions for each gene examined.

Because lens development is proceeding rapidly at p7 and is complete at p14 at eye opening, we identified 20 genes that, according to the array analysis, were significantly differentially expressed (*P* < 0.05) by at least 2-fold between p7 and p14. These genes are listed in Table 4. We used semi-quantitative RT-PCR in order to investigate the differential expression of these genes and, given the reduced amount of RNA required for RT-PCR as opposed to arrays, we also expanded the range of stages and looked at expression in Nb mouse lenses and 4 wk old mouse lenses in addition to p7 and p14 (Fig. 2). Differential expression was confirmed if shown in all three separate biological repeats. Confirmation rates were low (5/20; 25%), indicating that these arrays are excellent with regard to indicating gene expression, but less efficient at identifying differential expression. The genes confirmed as up-regulated at p14 compared to p7 were *Hd*, *Mdm2*, *Dffa*, *galectin-3* and *Mcl-1.* However, all genes except one expressed at background + 2SD using the arrays were also expressed as determined using RT-PCR (19/20, 95%; Fig. 2).

**Fig. 3:**
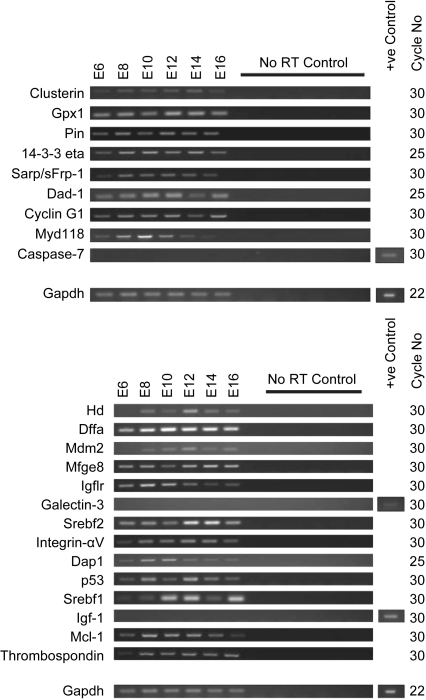
Semi-quantitative PCR results for the chicken homologues of selected apoptosis genes (where appropriate homologues can be identified). A. Chick homologues of genes with highest expression according to the mouse apoptosis arrays. B. Chick homologues of genes showing differential expression according to the mouse arrays. GAPDH was used as a loading control, no RT controls were included in the PCR reaction. Caspase-7, Galectin-3, and Igf-1 were not expressed; a positive control (whole 5 day chick embryo cDNA) was used to confirm that primers for these genes were working in the PCR reaction, confirming apparent lack of expression in the lens at the cycle numbers used. Results are representative of three repetitions for each gene examined.

To determine consistency of expression, hence biological relevance, by cross-species comparison, RT-PCR was used to examine the expression of the chick homologues of the most-highly expressed and/or potentially differentially regulated genes identified above (Fig. 3). From E6 to E16 (stages just prior to and just after the beginning of the major period of lens fibre cell organelle loss in chick lens fibres at E12), 9 of the most-highly expressed genes were also expressed in the chick embryo lens (Fig. 3A) as were 12 of the potentially differentially regulated genes (Fig. 3B). Galectin-3 and Igf-1 could not be amplified from any stage in the chick, despite bands in whole embryo cDNA positive controls. Chick homologues for the remaining genes could not be identified.

### 3.3 Protein expression studies: Western blotting and immunocytochemistry

**Fig. 4:**
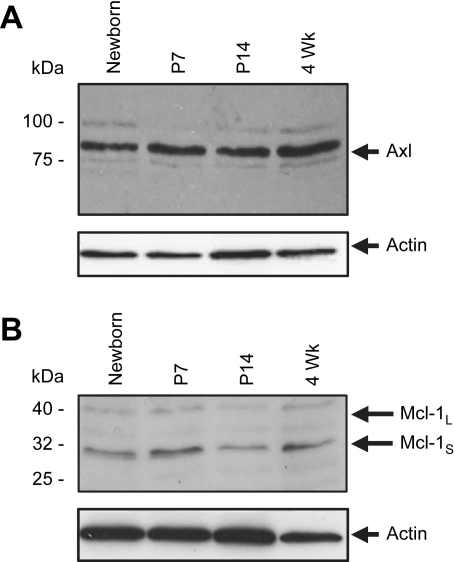
Western blotting results for Axl and Mcl-1 in the mouse lens. (A) Axl expression is observed at all stages examined at approximately 80 kDa. (B) Two bands for Mcl-1 are observed at all stages examined, a short form at approximately 32 kDa and a long form at approximately 40 kDa. In each case, the membranes were stripped and reprobed with an actin antibody to visualise loading of protein in each lane. Results are representative of three repetitions. See Supplementary Fig. 4 for bar graphs of mean normalised densitometry for these data.

Western blotting was used to confirm expression at the protein level of several genes identified using the mouse arrays in the mouse lens. Axl at 80 kDa had a fairly constant level of expression through Nb to 4 wk with slightly higher expression at Nb according to the densitometry data (Fig. 4A; Supplementary Fig. 4A). The short (pro-apoptotic) form of the blc-2 family member Mcl-1_S_ had highest expression at Nb and p7, with lower expression at p14 and 4 wk, while expression of the anti-apoptotic long Mcl-1_L_ was low at all stages examined, particularly at P14 and 4 wk by densitometry (Fig. 4B; Supplementary Fig. 4B and C).

**Fig. 5:**
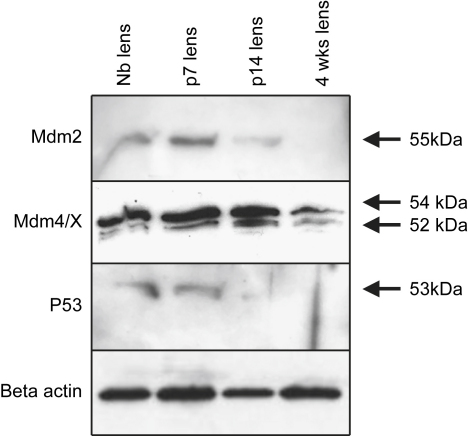
Western blots demonstrating expression of Mdm2, Mdm4/X and p53 in the postnatal mouse lens at newborn (Nb), p7, p14 and 4 wk (wk). At the protein level, expression of both Mdm2 and p53 is maximal at Nb-p7 and tails off thereafter. Mdm4/X exhibits upper (54 kDa; stronger) and lower (52 kDa; fainter) doublet bands at all stages examined. The 54 kDa band has a constant level of expression throughout the stages examined, while expression of the 52 kDa band peaks at p7–p14. The membranes were stripped and reprobed with an actin antibody to visualise loading of protein in each lane. Results are representative of three repetitions for each protein examined. See Supplementary Fig. 6 for bar graphs of mean normalised densitometry for these data.

Given the primary relevance of the p53 pathway to apoptosis, we elected to focus the remaining studies on Mdm2 and p53. Mdm2 was differentially expressed; moreover expression of both genes was confirmed in both mouse and chick by RT-PCR (see Figs. 2 and 3). The related gene MdmX/4 was not present on the array, but expression was demonstrated when tested by RT-PCR, so this gene was also examined further. Western blotting was used to confirm expression of all three proteins (p53, Mdm2, and Mdm4/X; Fig. 5; Supplementary Fig. 6). Mdm2 expression at approximately 55 kDa was detected in the Nb lens, peaked at p7, was reduced in expression at p14 and at 4 wk (Fig. 5; Supplementary Fig. 6A). Mdm4/X was present as a doublet at all stages examined: an intense upper band at approximately 54 kDa and a fainter lower band at approximately 52 kDa. The 54 kDa band had a constant level of expression throughout the stages examined, while expression of the 52 kDa band peaked at p7–p14 using densitometry (Fig. 5; Supplementary Fig. 6B and C). Expression levels of p53 were highest at Nb and p7, diminishing in intensity thereafter at p14 and 4 wk (Fig. 5; Supplementary Fig. 6D).

**Fig. 6:**
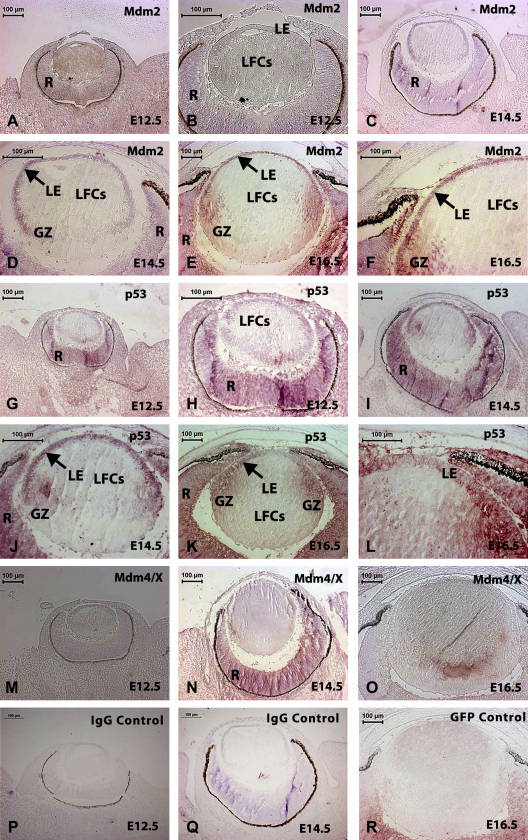
Immunocytochemical examination of spatio-temporal pattern of expression of Mdm2, p53 and Mdm4/X during mouse lens development. (A–F) Mdm2 expression. (A, B) E12.5 (higher and lower magnification), the lens did not express significant amounts of Mdm2. (C, D) E14.5 (higher and lower magnification), immature lens fibres in the germinal zone (GZ) and the lens epithelium (LE) showed strong Mdm2 expression. Staining was associated with nuclei. The retina (R) was also positive for Mdm2 immunoreactivity. (E, F) E16.5 (higher and lower magnification), Mdm2 was expressed in the lens epithelium (LE) and in peripheral lens fibre cells (LFCs) associated with the germinal zone (GZ) and was expressed in the nuclei of cortical lens fibre cells (LFCs). Mdm2 expression was lost as mature lens fiber cells (LFCs) differentiated. (G–L) p53 Expression. (G, H) E12.5 (lower and higher magnification). Moderate staining was observed in the lens epithelium (LE) and the immature lens fiber cells (arrow). (I, J) E14.5 (lower and higher magnification). p53 immunoreactivity was seen in the lens epithelium (LE) and in immature lens fibre cells (LFCs) in the germinal zone (GZ) region. There was some staining associated with nuclei of both the lens epithelial cells and lens fibre cells in a similar pattern as that seen for Mdm2 and in the retina (R). (K, L) E16.5 (lower and higher magnification), p53 expression was maintained in the lens epithelium (LE) and the immature lens fibres in the germinal zone (GZ). However, unlike Mdm2, p53 staining was not primarily associated with nuclei of the lens epithelial cells or fibre cells, but appeared to be primarily cytoplasmic. (M–O) Mdm4/X expression. Mdm4/X was not significantly expressed in the mouse lens at these stages of development. There appeared to be above-background levels of expression in the retina (R) at E14.5. (P–R) Representative negative control sections using rabbit IgG and GFP at the same concentration as the experimental primary antibodies showed negligible staining in the lens at E12.5, E14.5 and E16.5 confirming the specificity of the antibodies used. Scale bars, 100 μm.

Using RT-PCR, as well as confirming expression of Mdm2, Mdm4/X and p53 at postnatal stages of mouse lens development and in the adult lens, we also demonstrated expression of these genes in the E12.5 mouse eye, the E14.5 lens and the E16.5 lens (Supplementary Fig. 5). We therefore examined expression of Mdm2, p53 and Mdm4 in the developing mouse lens from E12.5 to E16.5 using immunocytochemistry (Fig. 6). We also attempted to examine expression of these proteins in sections of postnatal mouse lenses at Nb, p7 and p14, but were unable to obtain significant staining (data not shown; the more sensitive Western blots do demonstrate expression at these stages; Fig. 5).

Using immunocytochemistry, the lens did not express significant amounts of Mdm2 at E12.5 (Fig. 6A and B), while at E14.5 (Fig. 6C and D), immature lens fibres in the germinal zone (in which lens epithelial cells are differentiating into lens fibre cells) were positive for Mdm2. The lens epithelium also exhibited strong Mdm2 expression. Staining was associated with nuclei in both the lens epithelium and the lens fibre cells. The retina was also positive for Mdm2 immunoreactivity at E14.5. At E16.5 (Fig. 6E and F), Mdm2 was expressed in the lens epithelium and in peripheral lens fibre cells associated with the germinal zone, and was expressed in the nuclei of cortical lens fibre cells. Mdm2 expression was lost as mature lens fibre cells differentiated. Moderately intense p53 staining was observed in the lens epithelium and the immature lens fibre cells at E12.5 (Fig. 6G and H). At E14.5, p53 immunoreactivity was seen in the lens epithelium and in immature lens fibres in the germinal zone (Fig. 6I and J). There was also some staining at this stage associated with nuclei of both the lens epithelial cells and lens fibre cells in a similar pattern as that seen for Mdm2 and in the retina. At E16.5, p53 expression was maintained in the lens epithelium and the immature lens fibres in the germinal zone (Fig. 6K and L). However, unlike Mdm2, p53 staining at this stage was not primarily associated with nuclei of the lens epithelial cells or fibre cells, but appeared to be primarily cytoplasmic. Mdm4/X expression was not significantly expressed in the lens at these stages of development (Fig. 6M–O). There appeared to be above-background levels of Mdm4/X expression in the retina (R) at E14.5. Negative controls (Fig. 6P–R) using GFP as primary antibody or rabbit IgG at the same concentration as the experimental antibodies showed no positive staining at E12.5, E14.5, and E16.5 confirming the specificity of the staining observed.

**Fig. 7:**
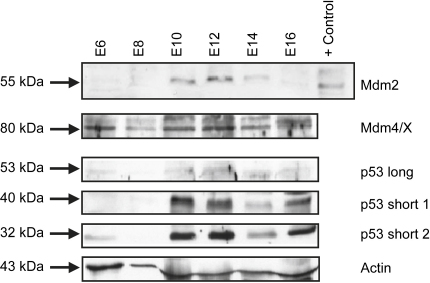
Western blot demonstrating expression of Mdm2, p53 and Mdm4/X in the chick lens at E6–E16. Expression of the 55 kDa Mdm2 band is faint at E6–E8, appears prominently at E10 and then peaks at E12, reducing in expression at E14–E16. The positive control lane for Mdm2 represents a Jurkat cell lysate provided with the antibody as a positive control. Mdm4/X at 80 kDa exhibits a fairly constant level of expression at all stages examined. Negligible levels of p53 at 53 kDa (p53 long) were detected at all stages examined, whereas shorter forms of p53 at approximately 40 kDa (p53 short 1) and 32 kDa (p53 short 2) were detected at highest levels from E10 to E12. The membranes were stripped and reprobed with an actin antibody to visualise loading of protein in each lane. Results are representative of three repetitions for each protein examined. See Supplementary Fig. 6 for bar graphs of mean normalised densitometry for these data.

**Fig. 8:**
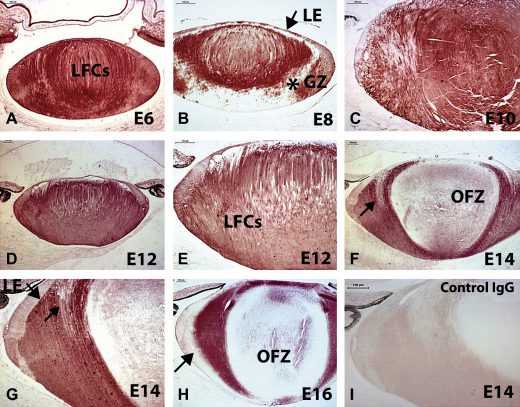
Immunocytochemical examination of spatio-temporal pattern of Mdm2 expression during chick lens development. (A) E6, Mdm2 was expressed throughout the lens. (B) E8, Mdm2 was expressed in the lens nucleus and lens epithelium (LE) as well as in the germinal zone (GZ), but was absent from cortical lens fibre cells (asterisk). (C) E10, Mdm2 expression was present throughout the lens fibre cells, but expression was beginning to diminish in the lens epithelium (LE, arrow). (D, E) E12 (lower and higher magnification). Mdm2 expression was present throughout the lens fibre cells (LFCs), but the expression in the lens epithelium was fainter. (F, G) E14 (lower and higher magnification). The outer cortical lens fibre cells showed strong Mdm2 expression, but the Mdm2 expression was lost from the lens epithelium and the nuclear fibre cells, coinciding with formation of an organelle free zone (OFZ). There was a small region close and anterior to the OFZ, which did not consistently stain significantly with the Mdm2 antibody (G; arrow). The lack of staining in the lens epithelium (LE) is also highlighted. The area anterior and close to the OFZ is also highlighted (arrow). (H) E16. Mdm2 was expressed in the outer cortical lens fiber cells, but was reduced in intensity in the lens epithelium as well as the outermost cortical fiber cells (arrow). (I) E14, rabbit IgG (IgG) control used at the same concentration as the anti-Mdm2 antibody. Magnification bars, 100 μm.

**Fig. 9:**
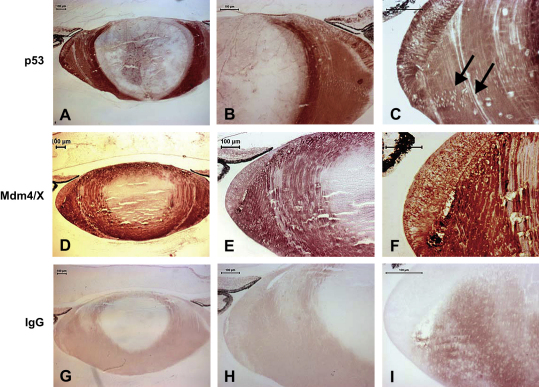
Immunocytochemical localization of MdmX, and p53 in the chick E14 lens. (A–C) p53 Staining is localised to the lens epithelium cells and the lens fibres cells in the outer lens cortex. More mature fibre cells deeper in the lens in the OFZ showed low to undetectable levels of p53 staining. p53 staining was absent from nuclei of the LFCs (arrowheads). (D–F) MdmX was expressed at relatively low levels in the lens epithelium and in cortical lens fibre cells, but was absent from the OFZ. (G–I) Rabbit IgG control used at the same concentration as the anti-p53 and anti-Mdm4/X antibodies. Magnification bars, 100 μm.

In order to check that cross-species expression was also occurring at the protein level, we examined the chick embryo lens using Western blotting and immunocytochemistry with the anti-mouse Mdm2, Mdm4/X and p53 antibodies (Figs. 7–9; Supplementary Fig. 7). Westerns revealed expression of an Mdm2-positive band at 55 kDa that was faint at E6–E8, peaked in intensity at E10–E12 and was reduced in expression from E14 to E16 (Fig. 7; Supplementary Fig. 7A). Mdm4/X gave a positive band at 80 kDa, which had a fairly constant expression throughout the stages examined (Fig. 7B; Supplementary Fig. 7B). Using the antibodies to p53, expression of a band at 53 kDa in the chick lens samples was low and relatively unaltered across all the stages examined (Fig. 7; Supplementary Fig. 7C), but we did detect expression of two bands at lower molecular weight 40 and 32 kDa respectively (p53 short 1 and p53 short 2) representing short forms of chicken p53, which both peaked in expression levels at E10 and E12 (Fig. 7 and Supplementary Fig. 7C and D).

Using immunocytochemistry, Mdm2 was expressed throughout the lens at E6 (Fig. 8A). At E8, Mdm2 was expressed in the lens nucleus and lens epithelium as well as in the germinal zone, but was absent from a band of cortical lens fibre cells (Fig. 8B; asterisk). At E10, Mdm2 expression was present throughout the lens fibre cell mass, but expression was beginning to diminish in the lens epithelium at this stage (Fig. 8C). At E12, Mdm2 expression was present throughout the lens fibre cell compartment, but the expression in the lens epithelium became fainter (Fig. 8D and E). At E14, the cortical lens fibre cells showed strong Mdm2 expression except for a small band close to and anterior to the organelle free zone (OFZ; Fig. 8F and G; arrows). Furthermore, Mdm2 expression was lost from the lens epithelium and the nuclear fibre cells, coinciding with formation of the OFZ (Fig. 8F and G). At E16, Mdm2 was expressed in the outer cortical lens fibre cells, but was reduced in intensity in the lens epithelium as well as the outermost cortical fibre cells (Fig. 8H). The rabbit IgG control, used at the same concentration as the anti-Mdm2 antibody, was negative for staining (Fig. 8I).

Given that Western blotting also gave positive signals for Mdm4/X and short forms of p53 in the chick embryo lens, immunostaining for p53 and Mdm4/X was also carried out on the chick embryo lens at E14 (Fig. 9) and compared to Mdm2 staining at this stage (Fig. 8F and G). The spatio-temporal pattern of expression of p53 was similar to Mdm2 and Mdm4/X, but with two notable differences; namely, that p53 staining was intense in the lens epithelium and was absent from lens fibre cell nuclei in the cortex of the lens (Fig. 9C; arrows). Mdm4/X had a similar pattern of expression to Mdm2, both being expressed at low levels in the lens epithelium and being intensely expressed in the cortical lens fibre cells (Fig. 9D–F; compare with Fig. 8F and G); IgG controls, at the same concentration as the experimental antibodies used were negative for staining, thereby confirming antibody specificity (Fig. 9G–I).

## 4 Discussion

In this study, we have carried out a thorough initial screen of apoptosis gene expression in the postnatal mouse lens using nylon arrays on which cDNAs representing 243 apoptosis genes were printed. We investigated apoptosis gene expression at two time points, p7 and p14, in order to determine which apoptosis genes were expressed at above-background levels at either or both stages and also to identify potentially differentially regulated genes. In support of the suggestion that the apoptosis signalling pathway has a significant role in lens development, 161 genes were expressed above-background levels + 2SDs of background. All genes expressed at p7 were also expressed at p14. Ninety five percent of genes tested by RT-PCR were indeed expressed in the lens. 5 genes were also identified correctly as being up-regulated at p14. Furthermore, cross-species conservation of expression of the majority of these genes was confirmed during chick lens development. Finally, since a number of members of the p53 signalling pathways were identified, we decided to focus in further experiments on the spatio-temporal pattern of expression of p53 and Mdm2 and the related molecule Mdm4/X. This is the first study to comprehensively investigate the spatio-temporal pattern of expression of p53, Mdm2 and Mdm4/X during lens development and, as such, implicates the p53 pathway in this process.

### 4.1 Overview of function of a selection of the genes identified from the array

Death-domain-associated protein (Daxx) was originally identified as a protein demonstrating specific binding to the death domain of the transmembrane death receptor FAS and was thought to be involved in the promotion of FAS-induced apoptosis (Yang et al., 1997). However, homozygous deletion of Daxx results in embryonic lethality, with widespread apoptosis observed in Daxx-deficient embryos (Michaelson et al., 1999). This suggests that Daxx plays an important role in embryonic development. This protein has also been shown to play a role in repression of transcriptional target genes (Michaelson and Leder, 2003). In this study, RNAi was used in various cell lines (HeLa, U2OS and 293 cells) to prevent the expression of Daxx and cells showed increased apoptosis, suggesting an anti-apoptotic role for Daxx, while transcriptional repression was also observed to decrease. Daxx is also involved in the p53/Mdm2 pathway (see below). Downregulation of Daxx decreases Mdm2 expression levels and Daxx enhances the E3 activity of Mdm2 towards p53 (Tang et al., 2006).

The exact physiological function of the normal Huntington disease (Hd) protein Huntingtin has yet to be elucidated. The mutant form of the *Huntingtin* gene, containing a CAG expansion region in the first exon, causes a progressive neurodegenerative disorder (Reddy et al., 1999). Huntingtin interacts with a wide range of proteins, including caspase-3, and it has been proposed to play a role in both membrane trafficking and apoptosis (Harjes and Wanker, 2003). Furthermore, there is recent evidence that expression of a mutant Huntingtin fragment in the lens results in protein aggregation and cataract formation (Muchowski et al., 2008). As far as we are aware, the current study is the first to demonstrate expression of the native *Huntingtin* gene in the lens during development. In preliminary studies (Geatrell et al., unpublished results), we quantified the size of the OFZ in the p2 mouse lens in Huntington mutant mice (containing an extended 150 bp CAG repeat in exon 1 of the *Huntingtin* gene; provided by Dr Leslie Jones, Cardiff University), compared to wild type and heterozygous mice. We could discern no effects of the mutant gene on the size of the OFZ in the lens. However, further studies are required to investigate the potential roles of Huntingtin in the lens during development and ageing. Indeed, the lens may be a particularly suitable and amenable model system in which to investigate the physiological roles of Huntingtin.

DNA fragmentation factor (DFF) is a heterodimer composed of 40 kDa and 45 kDa subunits (Liu et al., 1997). Caspase-3 cleaves the 45 kDa subunit (DFFA) at two sites to generate an active factor, resulting in DNA fragmentation without any further requirement for caspase-3 or other cytosolic proteases. We previously showed that DFFA is cleaved in the chick lens during organelle degradation (Wride et al., 1999), although this could be a caspase-independent process, as cleavage still occurred in the presence of general caspase inhibitor Boc-D-FMK.

Galectin-3 (gal-3) has been localised to the plasma membrane of ovine lens fibre cells where an interaction with MP20, an intrinsic membrane protein, was observed (Gonen et al., 2000). Expression of gal-3 in the human, mouse and rat lens has since been identified (Dahm et al., 2003). In human lenses, highest expression was observed during embryonic stages of development, although it continued to be expressed in adult lenses in both epithelial cells and early differentiating fibre cells (Dahm et al., 2003). Its expression was seen to decrease with maturation of the lens fibre cells; with no expression detected in mature lens fibres. The observations of the spatio-temporal expression of gal-3 lead to the suggestion that this molecule could play a role in cell–cell interactions and the differentiation of fibre cells. Gal-3 is also thought to play an anti-apoptotic role; a high level of both functional and structural similarity between gal-3 and Bcl-2 has been observed and gal-3 has been shown to prevent apoptosis induced by staurosporine in a human cell line (Yang et al., 1996).

Axl, a receptor tyrosine kinase, is expressed in both the bovine and rat lens epithelium (Valverde et al., 2004). Gas6 ligand was present in the aqueous humor and had both mitogenic and anti-apoptotic roles. It was proposed from this finding that the Gas6/Axl interaction could play a role in regulating the normal growth of lens epithelial cells.

Myeloid cell leukaemia-1 (Mcl-1) is a member of the Bcl-2 family of proteins and is predominantly localised in the mitochondrial membrane (Yang et al., 1995). Two isoforms of Mcl-1 have been identified; a short isoform, containing only a BH3 domain, which is pro-apoptotic (Bae et al., 2000) and the originally identified long form, containing Bcl-2 homology domains 1, 2 and 3, shown to be anti-apoptotic (Kozopas et al., 1993). The long and short isoforms are capable of forming heterodimers and the balance between the two isoforms could determine the fate of the cells expressing both proteins. The long isoform interacts with other pro-apoptotic Bcl-2 family members, but not with anti-apoptotic members (Bae et al., 2000).

Cyclin-dependent kinase 4 (cdk4) belongs to a family of serine/threonine protein kinases which are essential for the progression of the cell cycle (Sherr, 1993). The kinase activity of this protein is regulated by a member of the cyclin family, cyclin D. Cdk4 has a role in the regulation of the G1/S transition (Sherr, 1993). Both the mRNA and protein for cdk4 have previously been identified in both the epithelial and fibre cells of the rat lens during development (E16 to p8), alongside other members of the same family (Gao et al., 1999). Other members of the cyclin-dependent kinase family are involved in the process of primary fibre cell denucleation in the embryonic chick lens (He et al., 1998) so the detection of cdk4 here in both the mouse and chick embryo lens could suggest a role for cdk4 in this process as well.

Death associated protein 1 (Dap1) is a small proline rich protein shown to be located in the cytoplasm. Dap1 belongs to a family of 5 novel genes, shown to mediate cell death induced by interferon-γ (Levy-Strumpf and Kimchi, 1998). Dap1 interacts with the cytoplasmic death domain of TNF-R1 and overexpression of this protein induces apoptosis (Liou and Liou, 1999).

### 4.2 Expression of members of the p53 pathway in the mouse and chick lens

Since a number of members of the p53 signalling pathway were identified using the arrays, we elected to carry out a spatio-temporal analysis of expression of selected members of this family during both mouse and chick lens development.

P53 is a well-characterised tumour suppressor gene, which plays a role in a number of cellular processes including the response to DNA damage (Kastan et al., 1991), apoptosis (Shaw et al., 1992) and cell cycle progression (Kuerbitz et al., 1992). In the lens of adult mice, p53 is expressed in the lens epithelial cells of central and pre-germinative zones and in the lens fibre bow region (Pokroy et al., 2002). The role of p53 in lens cells has previously been examined using transgenic mice generated to express wild-type human p53. These mice developed microphthalmia due to apoptosis induction in differentiating lens fibre cells (Nakamura et al., 1995). Furthermore, it was also demonstrated that both p53-dependent and independent mechanisms may be active during lens development (Pan and Griep, 1995).

Mdm2 is involved in regulation of the cell cycle, apoptosis and tumourogenesis through its interactions with other proteins, including p53 and retinoblastoma 1 (Momand et al., 2000). Mdm2, has intrinsic E3 ligase activity and is the main inhibitor of p53, maintaining low levels of p53 expression in non-stressed cells by increasing the degradation of p53 by the 26S proteasome (Michael and Oren, 2003). Furthermore, p53 activity is altered by numerous post-translational modifications (Lavin and Gueven, 2006; Kruse and Gu, 2008); for example, acetylation is indispensable for p53 activation (Tang et al., 2008).

Here, we have provided an overview of the spatio-temporal patterns of expression of Mdm2, p53, and Mdm4/X during various stages of mouse and chick lens development (Figs. 6 and 8). In order to discuss similarities and differences in the pattern of expression of Mdm2 in the lens between the chick embryo and mouse embryo, it is necessary to compare the stages of lens development in the two species. In the mouse lens, organelle degradation begins in lens fibre cells at approximately E18.5, while in the chick it begins at approximately E12. Therefore, the mouse stages examined here (E12.5, E14.5 and E16.5) are prior to the onset of organelle degradation in the mouse and are the equivalent of approximately E6–E10 in the chick. It is apparent that there are species-specific differences in Mdm2 expression during lens development. In the mouse embryo lens at E12.5–E16.5, expression of Mdm2 is confined primarily to the lens epithelium, the germinal zone and nuclei of cortical lens fibre cells. In the chick, Mdm2 appears to have a more general pattern of expression, being expressed at E6 throughout the lens fibre cells and lens epithelial cells. At E8 in the chick embryo lens, expression of Mdm2 is localised to the lens epithelium and the germinal zone and this is similar to the pattern of expression in the mouse embryo (as described above). However, in the chick lens at E8, the expression of Mdm2 is unusual. It is not only expressed in the core lens fibre cells, but there is a lack of expression of Mdm2 in an intermediate band of cortical secondary fibre cells just outside the lens nucleus (Fig. 8B, asterisk). Since, at this stage, all lens fibre cells still contain their nuclei, it is possible that they reactivate expression of Mdm2 protein within this layer at E10 (at which stage, Mdm2 expression is throughout the fibre cell mass) and/or Mdm2 transcripts or protein synthesized in the germinal zone diffuse, or are transported, into the deeper cortical lens fibres. Future studies will examine this is more detail through a wider number of developmental stages and will also use *in situ* hybridisation in order to correlate Mdm2 mRNA with Mdm2 protein expression. It is also possible that Mdm2 protein is degraded transiently in these fibre cells and that this is dependent upon Mdm2 ubiquitination (by the ubiquitin ligase activity of Mdm2 itself or another ubiquitin ligase) and subsequent proteasomal degradation (Michael and Oren, 2003), thus allowing activation of p53 prior to the stages of lens fibre cell organelle loss. Intriguingly, this band of reduced Mdm2 immunoreactivity in the deeper cortical fibre cells also appears to be present in the anterior fibre cells just outside the OFZ at E14 (Fig. 8F, G; arrows). This could support the above suggestion of a transient loss of Mdm2 leading to an activation of p53 just outside the OFZ, implying an involvement of p53 in early events of lens fibre cell organelle loss. These possibilities require more thorough detailed analysis in future studies.

In the chick, following the onset of organelle degradation at E12, Mdm2 expression is progressively lost from the lens epithelium and becomes localised by E14–E16 to the cortical lens fibre cells (Fig. 8). P53 is expressed in the mouse lens in a similar pattern to Mdm2 in the lens epithelium and germinal zone, whereas Mdm4/X is not expressed significantly at comparable stages (Fig. 6). In the chick lens, we carried out immunocytochemistry at E14 using anti-mouse p53 and Mdm4/X antibodies and compared the pattern of expression with the Mdm2 staining at the same stage (Figs. 8 and 9). Both p53 and Mdm4/X are also expressed during chick lens development at this stage at which formation of the OFZ is occurring.

Finally, we also carried out Western blotting for Mdm2, Mdm4/X and p53 during both mouse and chick lens development (Figs. 5 and 7). Patterns of differential expression of these proteins were observed during both postnatal mouse lens development and at the comparable embryonic stages of chick lens development. Intriguingly, we identified two short forms of p53 at 40 kDa and 32 kDa during chicken lens development and short forms of Mdm4/X at approximately 54 kDa and 52 kDa respectively in the mouse lens. The short forms of chicken p53 have been identified previously in chicken lymphoblastoid cell lines and the 32 kDa form in particular was shown to be pro-apoptotic in these lines (Takagi et al., 2006). Regarding Mdm4/X, there is evidence for short forms of Mdm4/X, which have biological activity by modulating p53 through differential splicing of p53-binding domains (Rallapalli et al., 1999; Chandler et al., 2006). These splice variants have mostly been identified in the cancer field and there appears to be little, if anything in the literature about the roles of these potential splice variants/cleavage products of Mdm4/X in embryonic development. Furthermore, caspase-mediated cleavage of Mdm4/X resulted in detection of a 54 kDa protein on Westerns (Gentiletti et al., 2002); i.e. similar to the size of the proteins we detected here. Given the known activity of caspases during lens differentiation, it is likely that caspases are involved in regulating Mdm4/X activity and therefore modulating p53 signalling during lens development.

As far as we are aware, our results represent the first demonstration of the potentially pro-apoptotic short forms of chicken p53 in any developmental system. It is particularly intriguing that the short forms are particularly abundant at E10–E12 in the chick lens; stages at which organelle loss is beginning to occur. We did not identify comparable short forms of p53 in Western blots of the mouse lenses or, conversely, conclusive evidence of the short forms of Mdm4/X in the chick lens suggesting species-specific and/or developmental timing-related differences. Further studies are required to investigate the nature of such species differences and to define the expression and function of p53 and Mdm4/X splice variants/cleavage products in lens development.

It is also of interest that Mdm2 mRNA and protein levels do not always follow the same temporal pattern of expression. For example, in the mouse lens, when Western blotting experiments are compared to the RT-PCR data, the Mdm2 transcripts appear to be more abundant in the P14 lens than the P7 lens (Fig. 2). However, the Western blot suggests that the Mdm2 protein is more abundant in the P7 lens (Fig. 5). This is most-likely due to the balance between synthesis and degradation of Mdm2 protein, since Mdm2 protein can degrade itself through ubiquitin-mediated mechanisms (Michael and Oren, 2003). In this case, it is possible that even though more Mdm2 transcripts are present at p14 than at p7, Mdm2 protein degradation may be occurring to a greater extent at p14 than at p7. It is also of note that mRNA and protein levels also vary for p53 in which protein levels fall (Fig. 5; Supplementary Fig. 6), while transcript levels do not (Fig. 2). These data highlight the difficulties/complexities of correlating transcript levels with protein levels with regard to genes involved in modulating p53 signalling.

Thus, the spatio-temporal patterns of expression of Mdm2, p53, and Mdm4/X in the developing lens in both mouse and chick (albeit in subtly different patterns of expression at comparable stages) suggest a role for these oncoproteins in lens development. Given their spatio-temporal pattern of expression and known roles in regulation of cell proliferation and apoptosis signalling, we suggest that they have roles in lens fibre cell differentiation and this possibility merits further examination in further expression and functional studies in both species. In particular, it will be necessary to correlate spatio-temporal patterns of p53, Mdm2 and Mdm4/X expression in the developing lens with specific protein variants due to alternative splicing (e.g. the pro-apoptotic short forms of chicken p53 we have identified here), proteolytic cleavage (e.g. by caspases) and post-translational modifications (e.g. acetylation).

### 4.3 Concluding comments

This study represents an initial analysis of apoptosis gene expression in the postnatal mouse lens and provides an excellent resource for the lens research community for further investigation of the roles in the lens of the apoptosis genes identified. Furthermore, the results suggest a potential role for these apoptosis genes in the processes of lens differentiation and organelle degradation during lens development and/or in the regulation of classical apoptosis during lens development or postnatal maturation, ageing and possibly cataract.

Analysis of the spatio-temporal pattern of expression of these genes is an essential prerequisite for future studies. Indeed, building on the array studies, we have provided the first spatio-temporal analysis of expression of p53 pathway molecules (p53, Mdm2 and Mdm4/X) in both developing mouse and chick lenses. The developing lens presents an excellent model system in which a large number of fibre cells are maturing in a synchronised fashion. Therefore, it provides an excellent opportunity with which to study the normal function of members of the p53 signalling pathway in development. Thus, the results presented here pave the way for further studies investigating the functions of Mdm2, p53, and Mdm4/X and additional members of this pathway in lens development, physiology and potentially disease. Such studies will shed light on both normal lens development and on the normal developmental roles of Mdm2, p53, and Mdm4/X.

## Figures and Tables

**Table 1:** Sequence of mouse PCR primers for the genes identified from these arrays, annealing temperatures and the expected product size.

Accession number	Gene	Forward primer	Reverse prime	Annealing temperature (°C)	PCR product size (bp)
NM_001001303	Gapdh	ACCACAGTCCATGCCATCAC	TCCACCACCCTGTTGCTGTA	61	450
Genes shown to be differentially expressed between the two time points					
NM_010414	Hd	CTGCCACTCACCATTCTCACC	CCTCATCCCATTCCTCCTCTC	62	213
NM_008771	P2rx1	CTTGGCTATGTGGTGCGAGAG	TTGAAGAGGTGACGACGGTTT	62	233
NM_010044	Dffa	ACTTCCTCTGCCTTCCTTCCA	GCCACATTCTTCCACTTCACC	62	160
NM_010786	Mdm2	GCACACACACACACACACACA	AACATAGGCAACCACCAGGAA	61	240
NM_008594	Mfge8	CAACAACTCCCACAAGAAGAACA	AGAAGGTCGTCAGCCACAGAA	61	220
NM_010513	IGF1r	GCGGCGATGAAGAGAAGAAA	TCAGGAAGGACAAGGAGACCA	62	216
NM_009465	Axl	AAGAGCGATGTGTGGTCCTTC	GGCAGAGCCTTCAGTGTGTTC	61	248
NM_010705	Galectin-3	ACAGTGAAACCCAACGCAAAC	GCACAGACACACAACACACAAA	61	594
NM_009742	A1	ATTGCCCTGGATGTATGTGCT	GGTTCTCTCTGGTCCGTAGTGTT	61	219
NM_009870	Cdk4	CGACGCAGAGTGAGAAGAGG	TCAGGGAGGGAAGAAGACAGA	61	231
NM_009367	Tgf-β2	TTGGATGCTGCCTACTGCTTT	GCTTCGGGATTTATGGTGTTG	61	212
NM_011640	p53	GCTGGATAGGAAAGAGCACAGA	GGTTGAGGGCAAGAAATGGA	61	239
NM_146057	Dap1	CTGTGTCGCTAAGGAGGGATG	TTACAACGGGAGAAACTGACGA	62	121
XM_127995.	Srebf2	CAAGTCAGCAGCCAAGGAGAG	TCACAAATCCCACAGAGTCCA	61	233
NM_008402	Itg-αv	GGCTGCTGTGGAGATAAGAGG	GCCTTGCTGAATGAACTTGGA	61	162
NM_007829	DAXX	AAAGAAGCAACTGGGCTCTGG	GAGAAGCAGGGATGGAGAAGG	63	214
NM_008562	Mcl-1	ATTTCTTTCGGTGCCTTTGTG	AAACCCATCCCAGCCTCTTT	59	144
NM_010512	IGF-1	CTCTGCTTGCTCACCTTCACC	CACTCATCCACAATGCCTGTCT	63	176
NM_011480	Srebf1	TGGCTTGGTGATGCTATGTTG	AGGGAACTGTGTGTGTTTCTGG	61	150
NM_011580	Thrombospondin	CTGTGACCCTGGACTTGCTGT	AGTATCCCTGAGCCCTTGTGG	64	203

**Table 2:** Chick PCR primers for homologous genes of differentially regulated genes, including accession numbers of the sequence from which the primers were designed, annealing temperatures and expected product sizes. Genes for which a sequence could not be identified are highlighted in bold.

Accession number	Gene	Forward primer	Reverse primer	Annealing temperature (°C)	PCR product size (bp)
NM_001001303	Gapdh	GGAGAAACCAGCCAAGTATGATG	AAAGGTGGAAGAATGGCTGTCA	61	138
Genes shown to be differentially expressed between the two time points					
XM_420822	Hd	CCAGAAGGAGGTGGTGGTGT	AACAGGGCGAAGGGAAGAAG	62	250
–	**P2rx1**	Gene sequence not identified	–	–	–
XM_417610	Dffa	CTTGCCCAGAATCAAACCAAA	CGTGTCAACCACATCCATCTC	61	195
XM_416084	Mdm2	AACTGGTGCCGTCCTAATCT	TAATGTATGGTGGCTGGGTTG	59	148
XM_413867	Mfge8	GGAAGATGAGGCTGAGTGGTG	GCTGTGATGGGAGGGTCAAA	62	208
NM_205032	IGF1r	AAGTGCTCCGCTTTGTGATG	GAGGCTTGTTCTCTTCGCTGT	61	204
–	**Axl**	Gene sequence not identified	–	–	–
NM_214591	Galectin-3	CAGTTCCTCATTGTGCTTGG	GGACAGGGATTTGGTGTTAGG	59	165
–	**A1**	Gene sequence not identified	–	–	–
–	**Cdk4**	Gene sequence not identified	–	–	–
NM_001031045	Tgf-β2	CGGAAGGAGGAGGAAGAGGA	GAGGGAAGAAGTGATGGCAGA	62	325
NM_205264	p53	CGCTATGAGATGCTGAAGGAGA	CGTGGCTGAAGGGAAATGG	62	237
NM_001031003	Dap1	CACCAGCAGATTCAGGACAAA	TGCGTAAGGTAGGAACACATAGAG	61	345
XM_416222	Srebf2	GTGCCTCTCCTTCAACCCTTT	ATCATCCAGCCAAACCATCC	62	246
NM_205439	Itg-αV	TTGATTGTTGGAGCCTTTGGT	CTTTCCTTTGCCATCTGCTTT	60	189
–	**DAXX**	Gene sequence not identified	–	–	–
XM_001233734	Mcl-1	GAGGGCTTTGTTGACTTCTTCC	TCCACTTTGCCTTTCTCTCCT	61	178
NM_001004384	IGF-1	GATGCTCTTCAGTTCGTATGTGG	GCAGATTTAGGTGGCTTTATTGG	61	176
NM_204126	Srebf1	GCAGAAGAGCAAGTCCCTCAA	GTCGGCATCTCCATCACCTC	63	105
XM_421205	Thrombospondin	GGGTGAAGCAAGAGAAACCAA	CGCAAAGCAGGGATTAGACA	60	250

**Table 3:** The ten most-highly expressed genes at P7 and P1. For a complete list of all genes printed on the array, see Supplementary Table 1. For a complete list of all genes expressed above background, plus 2SDs of background at both p7 and p14, please see Supplementary Tables 2 and 3.

Gene name	Accession number	Normalised band intensity P7	Normalised band intensity P14	Gene family
*Clusterin*	NM_013492	75.36	63.25	Apoptosis-related factors
*Gpx1*	NM_008160	55.09	48.46	Apoptosis-related factors
*Pin*	NM_019682	50.64	42.09	Apoptosis-related factors
*14-3-3 eta*	NM_011738	47.45	28.95	Signal transduction
*SARP-2/sfrp-1*	NM_013834	39.34	56.28	Apoptosis-related factors
*Dad-1*	NM_010015	32.63	27.49	Apoptosis-related factors
*Cyclin-G1*	NM_009831	28.61	51.28	Cell cycle regulators
*Myd118*	NM_008655	28.61	20.37	Signal transduction
*Mts-1*	NM_011311	23.74	28.27	Apoptosis-related factors
*Caspase-7*	NM_007611	18.92	18.79	Caspases and regulators

**Table 4:** Differentially expressed genes, p7 and p14. Normalisation was carried out using the housekeeping gene method (see text for details). This table lists the genes shown to have a 2-fold or greater difference in expression between P7 and P14 and a *t*-test *p* < 0.05.

Gene name	Accession number	Mean normalised expression P7	Mean normalised expression P14	Fold difference	*p*-Value
*P2rx1*	NM_008771	2.05	18.05	8.81	1.60E−05
*Hd*	NM_010414	2.97	21.96	7.34	4.50E−06
*Icad/Dffa*	NM_010044	3.91	15.03	3.84	0.0002
*Mdm2*	NM_010786	4.78	16.8	3.51	4.60E−07
*Mfge8*	NM_008594	2.52	7.89	3.13	0.0004
*IGF1r*	NM_010513	2.33	6.84	2.93	0.0001
*Axl*	NM_009465	2.85	8.27	2.9	0.0017
*Galectin-3*	NM_010705	2.49	6.67	2.68	0.0002
*A1*	NM_009742	2.46	6.53	2.66	0.0014
*Cdk4*	NM_009870	2.79	7.2	2.58	0.0005
*Srebf2*	XM_127995	4.62	11.86	2.57	0.0062
Tgfβ2	NM_009367	3.83	9.32	2.43	2.50E−06
Integrin-aV	NM_008402	6.56	14.61	2.23	0.0002
*Dap1*	NM_146057	7.56	16.85	2.23	0.0013
*Trp53/p53*	NM_011640	3.17	7.01	2.21	0.0101
*Srebf1*	NM_011480	2.49	5.43	2.18	0.007
*IGF-1*	NM_010512	3.52	7.65	2.17	0.035
*Mcl-1*	NM_008562	3.79	8.04	2.12	5.90E−06
*Thrombospondin*	NM_0011580	2.99	6.04	2.02	0.003
*Daxx*	NM_007829	2.14	4.32	2.01	0.024

## Appendix Supplementary data

Supplementary Fig. 1 Phosphoimage of an array hybridised with P7 radiolabelled RNA. Examples of positive spots are provided, including genomic DNA spots for orientation of the array as well as housekeeping genes and apoptosis genes (caspase-7 and 14-3-3 eta).

Supplementary Fig. 2 Reproducibility of array results. Scatter plots (A–E) were produced by plotting the normalised signal intensities from one array against another. Array results were compared between arrays that had been treated identically (i.e. fresh array compared with a fresh array, or a stripped array compared with a stripped array) or that had been treated differently (fresh versus stripped). Results from the arrays hybridised with P7 cDNA (A–C) or P14 cDNA (D, E) show that when arrays are compared with others that have been treated identically they have a high level of reproducibility. However, when the results were compared from two different conditions the trend-line did not dissect equal values on both the X and Y axes (C). The R2-value is shown on each graph. The closer this value is to 1, the greater the correlation between the two sets of data. Because of the variation observed when comparing stripped versus fresh arrays, the normalisation approach used was the housekeeping approach, since if one assumes linearity, one can account for the spots being fainter overall by scaling up (normalising) according to the housekeeping genes.

Supplementary Fig. 3 Standard curves for semi-quantitative PCR of chicken embryo lens. Standard curves were completed for three genes expressed at relatively high (GAPDH), intermediate (13-3-3 eta) and low levels (Mdm2) to determine the number of cycles to be used to ensure the PCR was in the linear phase of the reaction. The increase in band intensity with increasing cycles from 16–30 (shown underneath each graph) was used to produce the curve. –RT: no RT control. From these results, the cycle number used for each of these primer pairs was determined: GAPDH: 22 cycles; 14-3-3 eta: 25 cycles; Mdm2: 30 cycles.

Supplementary Fig. 4 Graphical representations of representative Western blots using densitometry normalised against the actin control at each stage examined for mouse Axl-1 and Mcl-1. Error bars represent standard deviation of the mean for a minimum of 3 replicates in each case. (A) Axl expression peaked at Nb and had a constant low level of expression thereafter to 4 wk. (B) The short (32 kDa; pro-apoptotic) form of the blc-2 family member Mcl-1_S_ had highest expression at Nb and p7, with lower expression at p14 and 4 wk, while expression of the long (40 kDa; anti-apoptotic) Mcl-1_L_ was low at all stages examined with marginally higher expression at Nb.

Supplementary Fig. 5 RT-PCRs reveal expression of Mdm2, Mdm4/X and p53 during embryonic stages of mouse lens development. PCRs were also carried out on cDNA prepared as described in Materials and Methods from E12.5 pooled mouse embryo whole eyes and on pooled lenses from E14.5, E16.5 as well as the post-natal stages examined previously. All three genes were differentially regulated at relatively high levels, compared to post-natal stages in the embryonic eyes/lenses. We were unable to obtain sufficient protein lysate for Western blotting from these stages of lens development.

Supplementary Fig. 6 Graphical representations of representative Western blots using densitometry normalised against the actin control at each stage examined for mouse Mdm2, Mdm4/X and p53. Error bars represent standard deviation of the mean for a minimum of 3 replicates in each case. (A) Mdm2 expression at approximately 55 kDa was detected in the Nb lens, peaked at p7 and was reduced at p14 to 4 wk. (B and C) Mdm4/X was expressed as a doublet with a higher (more intense) band at approximately 54 kDa (B) and a lower (fainter) band at approximately 52 kDa. The band at 54 kDa band had a relatively constant level of expression across all stages examined, while the lower band of the doublet was expressed at lower levels with a peak of expression at p7-p14. (D) Expression of p53 peaked at Nb becoming virtually undetectable at 4 wk.

Supplementary Fig. 7 Graphical representations of representative Western blots using densitometry normalised against the actin control at each stage examined for chick Mdm2, Mdm4/X and p53. Error bars represent standard deviation of the mean for a minimum of 3 replicates in each case. (A) The Mdm2-positive band at 55 kDa was faint at embryonic day (ED)6-ED8, peaked in intensity at ED10-ED12 and was reduced in expression from ED14 to ED16. (B) Mdm4/X at 80 kDa showed a level of expression at all stages with highest expression at ED16. (C) Expression of p53 at 53 kDa in the chick lens samples was low at all stages, but expression was marginally higher at ED6. (D) Expression of a 40 kDa p53-positive band peaked at ED10-ED12. (E) Expression of a 32 kDa p53-positive band peaked at ED10-ED12 was reduced at ED14 and increased again at ED16.

## References

[bib1] Ayala M., Strid H., Jacobsson U., Soderberg P.G. (2007). p53 Expression and apoptosis in the lens after ultraviolet radiation exposure. Investigative Ophthalmology and Vision Science.

[bib2] Bae J., Leo C.P., Hsu S.Y., Hsueh A.J.W. (2000). Mcl-1s, a splicing variant of the antiapoptotic Bcl-2 family member Mcl-1, encodes a proapoptotic protein possessing only the BH3 domain. Journal of Biological Chemistry.

[bib3] Bassnett S. (2002). Lens organelle degradation. Experimental Eye Research.

[bib4] Bassnett S. (2008). On the mechanism of organelle degradation in the vertebrate lens. Experimental Eye Research.

[bib5] Bassnett S., Beebe D.C. (1992). Coincident loss of mitochondria and nuclei during lens fiber cell-differentiation. Developmental Dynamics.

[bib6] Bassnett S., Mataic D. (1997). Chromatin degradation in differentiating fiber cells of the eye lens. Journal of Cell Biology.

[bib7] Brazma A., Hingamp P., Quackenbush J., Sherlock G., Spellman P., Stoeckert C., Aach J., Ansorge W., Ball C.A., Causton H.C., Gaasterland T., Glenisson P., Holstege F.C., Kim I.F., Markowitz V., Matese J.C., Parkinson H., Robinson A., Sarkans U., Schulze-Kremer S., Stewart J., Taylor R., Vilo J., Vingron M. (2001). Minimum information about a microarray experiment (MIAME)-toward standards for microarray data. Nature Genetics.

[bib8] Chandler D.S., Singh R.K., Caldwell L.C., Bitler J.L., Lozano G. (2006). Genotoxic stress induces coordinately regulated alternative splicing of the p53 modulators MDM2 and MDM4. Cancer Research.

[bib9] Congdon N.G. (2001). Prevention strategies for age related cataract: present limitations and future possibilities. British Journal of Ophthalmology.

[bib10] Cvekl A., Piatigorsky J. (1996). Lens development and crystallin gene expression: many roles for Pax-6. Bioassays.

[bib11] Dahm R. (1999). Lens fibre cell differentiation – a link with apoptosis?. Ophthalmic Research.

[bib12] Dahm R., Bramke S., Dawczynski J., Nagaraj R.H., Kasper M. (2003). Developmental aspects of galectin-3 expression in the lens. Histochemistry and Cell Biology.

[bib13] Dahm R., Gribbon C., Quinlan R.A., Prescott A.R. (1998). Changes in the nucleolar and coiled body compartments precede lamina and chromatin reorganization during fibre cell denucleation in the bovine lens. European Journal of Cell Biology.

[bib14] Danilova N., Sakamoto K.M., Lin S. (2008). p53 Family in development. Mechanisms of Development.

[bib15] Danilova N., Sakamoto K.M., Lin S. (2008). Role of p53 family in birth defects: lessons from zebrafish. Birth Defects Research C: Embryo Today.

[bib16] Faulkner-Jones B., Zandy A.J., Bassnett S. (2003). RNA stability in terminally differentiating fibre cells of the ocular lens. Experimental Eye Research.

[bib17] Francis P.J., Berry V., Bhattacharya S.S., Moore A.T. (2000). The genetics of childhood cataract. Journal of Medical Genetics.

[bib18] Francis P.J., Berry V., Moore A.T., Bhattacharya S. (1999). Lens biology: development and human cataractogenesis. Trends in Genetics.

[bib19] Gao C.Y., Rampalli A.M., Cai H., He H., Zelenka P.S. (1999). Changes in cyclin dependent kinase expression and activity accompanying lens fiber cell differentiation. Experimental Eye Research.

[bib20] Gentiletti F., Mancini F., D'Angelo M., Sacchi A., Pontecorvi A., Jochemsen A.G., Moretti F. (2002). MDMX stability is regulated by p53-induced caspase cleavage in NIH3T3 mouse fibroblasts. Oncogene.

[bib21] Gonen T., Donaldson P., Kistler J. (2000). Galectin-3 is associated with the plasma membrane of lens fiber cells. Investigative Ophthalmology and Vision Science.

[bib22] Graw J. (2004). Congenital hereditary cataracts. International Journal of Developmental Biology.

[bib23] Graw J., Loster J. (2003). Developmental genetics in ophthalmology. Ophthalmic Genetics.

[bib24] Gregg N.M., Banatvala J.E. (2001). Congenital cataract following German measles in the mother (Reprinted from Transactions Ophthalmological Society of Australia 3, pp. 35–46, 1942). Reviews in Medical Virology.

[bib25] Harjes P., Wanker E.E. (2003). The Hunt for Huntingtin function: interaction partners tell many different stories. Trends in Biochemical Sciences.

[bib26] Hawse J., Hejtmancik J., Huang Q.L., Sheets N., Hosack D., Lempicki R., Horwitz J., Kantorow M. (2003). Identification and functional clustering of global gene expression differences between human age-related cataract and clear lenses. Molecular Vision.

[bib27] Hawse J.R., Hejtmancik J.F., Horwitz J., Kantorow M. (2004). Identification and functional clustering of global gene expression differences between age-related cataract and clear human lenses and aged human lenses. Experimental Eye Research.

[bib28] He H.Y., Gao C., Vrensen G., Zelenka P. (1998). Transient activation of cyclin B/Cdc2 during terminal differentiation of lens fiber cells. Developmental Dynamics.

[bib29] Ireland M.E., Wallace P., Sandilands A., Poosch M., Kasper M., Graw J., Liu A., Maisel H., Prescott A.R., Hutcheson A.M., Goebel D., Quinlan R.A. (2000). Up-regulation of novel intermediate filament proteins in primary fiber cells: an indicator of all vertebrate lens fiber differentiation. The Anatomical Record.

[bib30] Ivanov D., Dvoriantchikova G., Pestova A., Nathanson L., Shestopalov V.I. (2005). Microarray analysis of fiber cell maturation in the lens. FEBS Letters.

[bib31] Kastan M., Onyekwere O., Sidransky D., Vogelstein B., Craig R.W. (1991). Participation of P53 protein in the cellular response to DNA damage. Cancer Research.

[bib32] Kozopas K.M., Yang T., Buchan H.L., Zhou B.P., Craig R.W. (1993). Mcl1, a gene expressed in programmed myeloid cell differentiation, has sequence similarity to Bcl-2. Proceedings of the National Academy of Sciences of the United States of America.

[bib33] Kruse J.P., Gu W. (2008). SnapShot: p53 posttranslational modifications. Cell.

[bib34] Kuerbitz S., Plunkett B., Walsh W., Kastan M. (1992). Wild-type P53 is a cell cycle checkpoint determinant following irradiation. Proceedings of the National Academy of Sciences of the United States of America.

[bib35] Kuwabara T., Imaizumi M. (1974). Denucleation process of the lens. Investigative Ophthalmology.

[bib36] Lavin M.F., Gueven N. (2006). The complexity of p53 stabilization and activation. Cell Death Differ.

[bib37] Lengner C.J., Steinman H.A., Gagnon J., Smith T.W., Henderson J.E., Kream B.E., Stein G.S., Lian J.B., Jones S.N. (2006). Osteoblast differentiation and skeletal development are regulated by Mdm2-p53 signaling. Journal of Cell Biology.

[bib38] Levy-Strumpf N., Kimchi A. (1998). Death associated proteins (DAPs): from gene identification to the analysis of their apoptotic and tumor suppressive functions. Oncogene.

[bib39] Liou M.L., Liou H.C. (1999). The Ubiquitin-Homology Protein DAP-1 associates with tumor necrosis factor receptor (p60) death domain and induces apoptosis. Journal of Biological Chemistry.

[bib40] Liu G., Terzian T., Xiong S., Van Pelt C.S., Audiffred A., Box N.F., Lozano G. (2007). The p53-Mdm2 network in progenitor cell expansion during mouse postnatal development. Journal of Pathology.

[bib41] Liu X.S., Zou H., Slaughter C., Wang X.D. (1997). DFF, a heterodimeric protein that functions downstream of caspase-3 to trigger DNA fragmentation during apoptosis. Cell.

[bib42] Mansergh, F.C., Hunter, S.M., Geatrell, J.C., Jarrin, M., Powell, K., Evans, M.J., Wride, M.A., 2008. Developmentally regulated expression of hemoglobin subunits in avascular tissues. International Journal of Developmental Biology, 52(7), 873–886. 10.1387/ijdb.082597fm 18956317

[bib43] Mansergh F.C., Wride M.A., Walker V.E., Adams S., Hunter S.M., Evans M.J. (2004). Gene expression changes during cataract progression in Sparc null mice: differential regulation of mouse globins in the lens. Molecular Vision.

[bib44] Marine J.C., Francoz S., Maetens M., Wahl G., Toledo F., Lozano G. (2006). Keeping p53 in check: essential and synergistic functions of Mdm2 and Mdm4. Cell Death Differ.

[bib45] McAlister Gregg N. (2001). Congenital cataract following German measles in the mother 1942. [classical article]. Reviews in Medical Virology.

[bib46] Michael D., Oren M. (2003). The P53-Mdm3 module and the uibiquitin system. Seminars in Cancer Biology.

[bib47] Michaelson J.S., Bader D., Kuo F., Kozak C., Leder P. (1999). Loss of Daxx, a promiscuously interacting protein, results in extensive apoptosis in early mouse development. Genes and Development.

[bib48] Michaelson J.S., Leder P. (2003). RNAi reveals anti-apoptotic and transcriptionally repressive activities of DAXX. Journal of Cell Science.

[bib49] Momand J., Wu H., Dasgupta G. (2000). MDM2-master regulator of the P53 tumor suppressor protein. Gene.

[bib50] Muchowski P.J., Ramsden R., Nguyen Q., Arnett E.E., Greiling T.M., Anderson S.K., Clark J.I. (2008). Noninvasive measurement of protein aggregation by mutant Huntingtin fragments or alpha-synuclein in the lens. Journal of Biological Chemistry.

[bib51] Nakahara M., Nagasaka A., Koike M., Uchida K., Kawane K., Uchiyama Y., Nagata S. (2007). Degradation of nuclear DNA by DNase II-like DNase in cortical fiber cells of mouse eye lens. FEBS.

[bib52] Nakamura T., Pichel J., Williams-Simons L., Westphal H. (1995). An apoptotic defect in lens differentiation caused by P53 is rescued by a mutant allele. Proceedings of the National Academy of Sciences of the United States of America.

[bib53] Nishimoto S., Kawane K., Watanabe-Fukunaga R., Fukuyama H., Ohsawa Y., Uchiyama Y., Hashida N., Ohguro N., Tano Y., Morimoto T., Fukuda Y., Nagata S. (2003). Nuclear cataract caused by a lack of DNA degradation in the mouse eye lens. Nature.

[bib54] Pan H., Griep A.E. (1995). Temporally distinct patterns of p53-dependent and p53-independent apoptosis during mouse lens development. Genes and Development.

[bib55] Pendergrass W., Penn P., Possin D., Wolf N. (2005). Accumulation of DNA, nuclear and mitochondrial debris, and ROS at sites of age-related cortical cataract in mice. Investigative Ophthalmology and Vision Science.

[bib56] Pendergrass W.R., Penn P.E., Possin D.E., Wolf N.S. (2006). Cellular debris and ROS in age-related cortical cataract are caused by inappropriate involution of the surface epithelial cells into the lens cortex. Molecular Vision.

[bib57] Piatigorsky J. (1981). Lens differentiation in vertebrates – a review of cellular and molecular-features. Differentiation.

[bib58] Pokroy R., Tendler Y., Pollack A., Zinder O., Weisinger G. (2002). P53 expression in the normal murine eye. Investigative Ophthalmology and Vision Science.

[bib59] Rallapalli R., Strachan G., Cho B., Mercer W.E., Hall D.J. (1999). A novel MDMX transcript expressed in a variety of transformed cell lines encodes a truncated protein with potent p53 repressive activity. Journal of Biological Chemistry.

[bib60] Reddy P.H., Williams M., Tagle D.A. (1999). Recent advances in understanding the pathogenesis of Huntington's disease. Trends in Neurosciences.

[bib61] Ruotolo R., Grassi F., Percudani R., Rivetti C., Martorana D., Maraini G., Ottonello S. (2003). Gene expression profiling in human age-related nuclear cataract. Molecular Vision.

[bib62] Segev F., Mor O., Segev A., Belkin M., Assia E.I. (2004). Downregulation of gene expression in the ageing lens: a possible contributory factor in senile cataract. Eye.

[bib63] Shaw P., Bovey R., Tardy S., Sahli R., Sordat B., Costa J. (1992). Induction of apoptosis by wild-type P53 in a human colon tumor-derived cell line. Proceedings of the National Academy of Sciences of the United States of America.

[bib64] Sherr C.J. (1993). Mammalian G1 cyclins. Cell.

[bib65] Takagi M., Takeda T., Asada Y., Sugimoto C., Onuma M., Ohashi K. (2006). The presence of a short form of p53 in chicken lymphoblastoid cell lines during apoptosis. Journal of Veterinary Medical Science.

[bib66] Tang J., Qu L.K., Zhang J., Wang W., Michaelson J.S., Degenhardt Y.Y., El-Deiry W.S., Yang X. (2006). Critical role for Daxx in regulating Mdm2. Nature Cell Biology.

[bib67] Tang Y., Zhao W., Chen Y., Zhao Y., Gu W. (2008). Acetylation is indispensable for p53 activation. Cell.

[bib68] Tendler Y., Panshin A., Weisinger G., Zinder O. (2006). Identification of cytoplasmic p53 protein in corneal epithelium of vertebrates. Experimental Eye Research.

[bib69] Toledo F., Wahl G.M. (2007). MDM2 and MDM4: p53 regulators as targets in anticancer therapy. International Journal of Biochemistry and Cell Biology.

[bib70] Valverde P., Obin M.S., Taylor A. (2004). Role of Gas6/Axl signaling in lens epithelial cell proliferation and survival. Experimental Eye Research.

[bib71] Wride M.A. (1996). Cellular and molecular features of lens differentiation: a review of recent advances. Differentiation.

[bib72] Wride M.A. (2000). Minireview: apoptosis as seen through a lens. Apoptosis.

[bib73] Wride M.A. (2007). Proteases in the development and diseases of the lens. Expert Review of Ophthalmology.

[bib74] Wride M.A., Mansergh F.C., Adams S., Everitt R., Minnema S.E., Rancourt D.E., Evans M.J. (2003). Expression profiling and gene discovery in the mouse lens. Molecular Vision.

[bib75] Wride M.A., Parker E., Sanders E.J. (1999). Members of the Bcl-2 and caspase families regulate nuclear degeneration during chick lens fibre differentiation. Developmental Biology.

[bib76] Wride M.A., Sanders E.J. (1998). Nuclear degeneration in the developing lens and its regulation by TNF alpha. Experimental Eye Research.

[bib77] Xiao W., Liu W., Li Z., Liang D., Li L., White L.D., Fox D.A., Overbeek P.A., Chen Q. (2006). Gene expression profiling in embryonic mouse lenses. Molecular Vision.

[bib78] Xiong S., Van Pelt C.S., Elizondo-Fraire A.C., Liu G., Lozano G. (2006). Synergistic roles of Mdm2 and Mdm4 for p53 inhibition in central nervous system development. Proceedings of the National Academy of Sciences of the United States of America.

[bib79] Yang R., Hsu D.K., Liu F. (1996). Expression of Galectin-3 modulates T-cell growth and apoptosis. Proceedings of the National Academy of Sciences of the United States of America.

[bib80] Yang T., Kozopas K.M., Craig R.W. (1995). The intracellular distribution and pattern of expression of Mcl-1 overlap with, but are not identical to, those of Bcl-2. Journal of Cell Biology.

[bib81] Yang X., Khosravi-Far R., Chang H.Y., Baltimore D. (1997). Daxx, a novel Fas-binding protein that activates JNK and apoptosis. Cell.

[bib82] Zandy A.J., Lakhani S., Zheng T., Flavell R.A., Bassnett S. (2005). Role of the executioner caspases during lens development. Journal of Biological Chemistry.

