# Supplementary figures and images for: Apoptosis gene profiling reveals spatio-temporal regulated expression of the p53/Mdm2 pathway during lens development
Source: Exp Eye Res. 2009 Jun 1;88(6):1137–51. doi: 10.1016/j.exer.2009.01.020 (PMC2706329; doi:10.1016/j.exer.2009.01.020)

**Supplementary Figure 1: Geatrell et al (2008)**

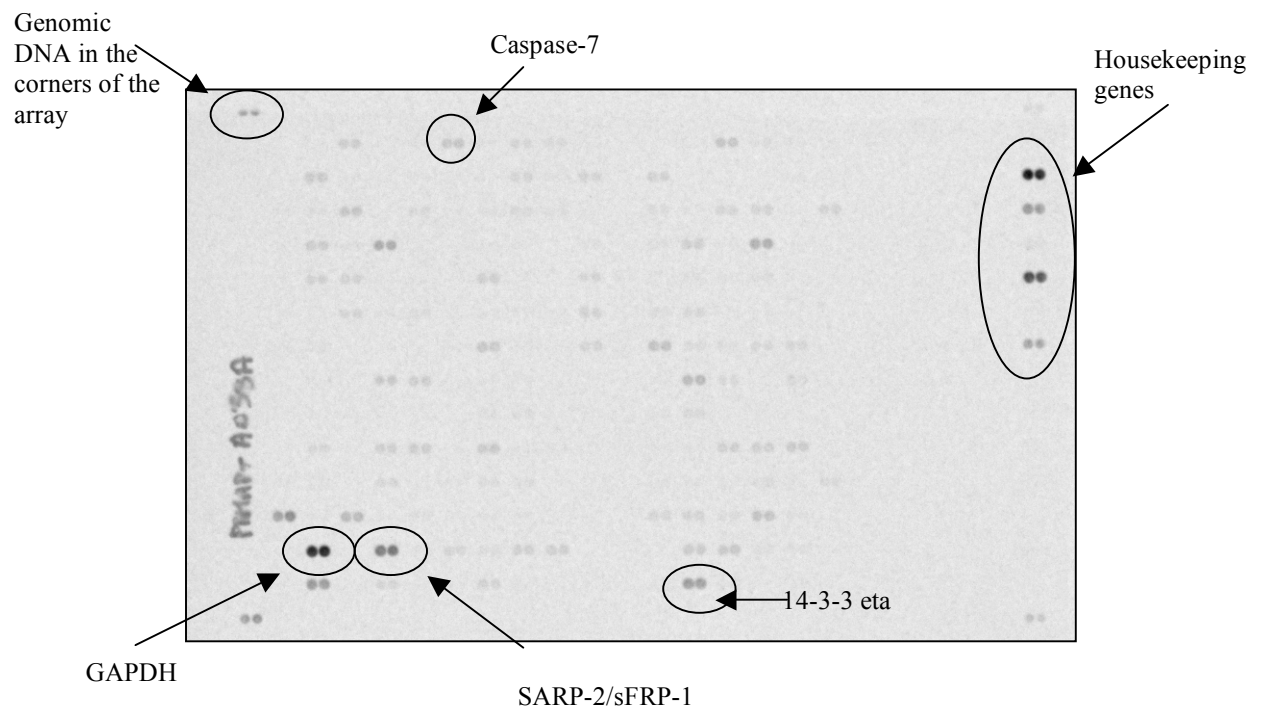

Supplement: Supplementary Fig. 1 — Phosphoimage of an array hybridised with P7 radiolabelled RNA. Examples of positive spots are provided, including genomic DNA spots for orientation of the array as well as housekeeping genes and apoptosis genes (caspase-7 and 14-3-3 eta). [file mmc1.pdf]

## Supplementary Figure 2: Geatrell et al (2008)

**A**

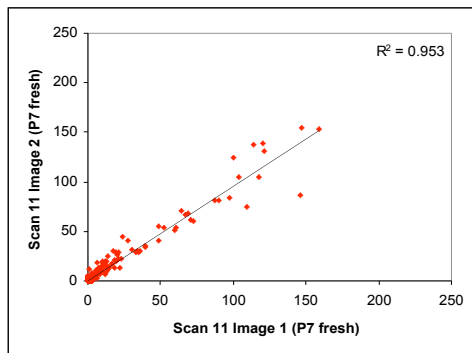

**B**

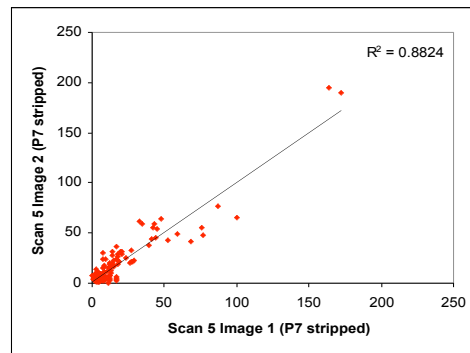

**C**

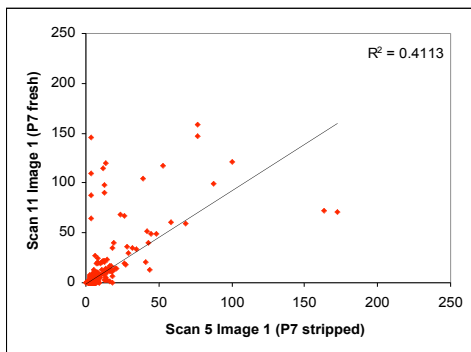

**D**

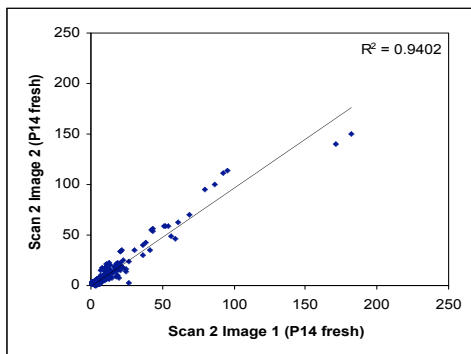

**E**

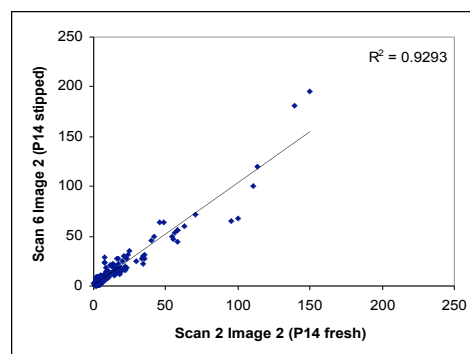

Supplement: Supplementary Fig. 2 — Reproducibility of array results. Scatter plots (A–E) were produced by plotting the normalised signal intensities from one array against another. Array results were compared between arrays that had been treated identically (i.e. fresh array compared with a fresh array, or a stripped array compared with a stripped array) or that had been treated differently (fresh versus stripped). Results from the arrays hybridised with P7 cDNA (A–C) or P14 cDNA (D, E) show that when arrays are compared with others that have been treated identically they have a high level of reproducibility. However, when the results were compared from two different conditions the trend-line did not dissect equal values on both the X and Y axes (C). The R2-value is shown on each graph. The closer this value is to 1, the greater the correlation between the two sets of data. Because of the variation observed when comparing stripped versus fresh arrays, the normalisation approach used was the housekeeping approach, since if one assumes linearity, one can account for the spots being fainter overall by scaling up (normalising) according to the housekeeping genes. [file mmc2.pdf]

Supplementary Figure 3: Geatrell et al (2008):

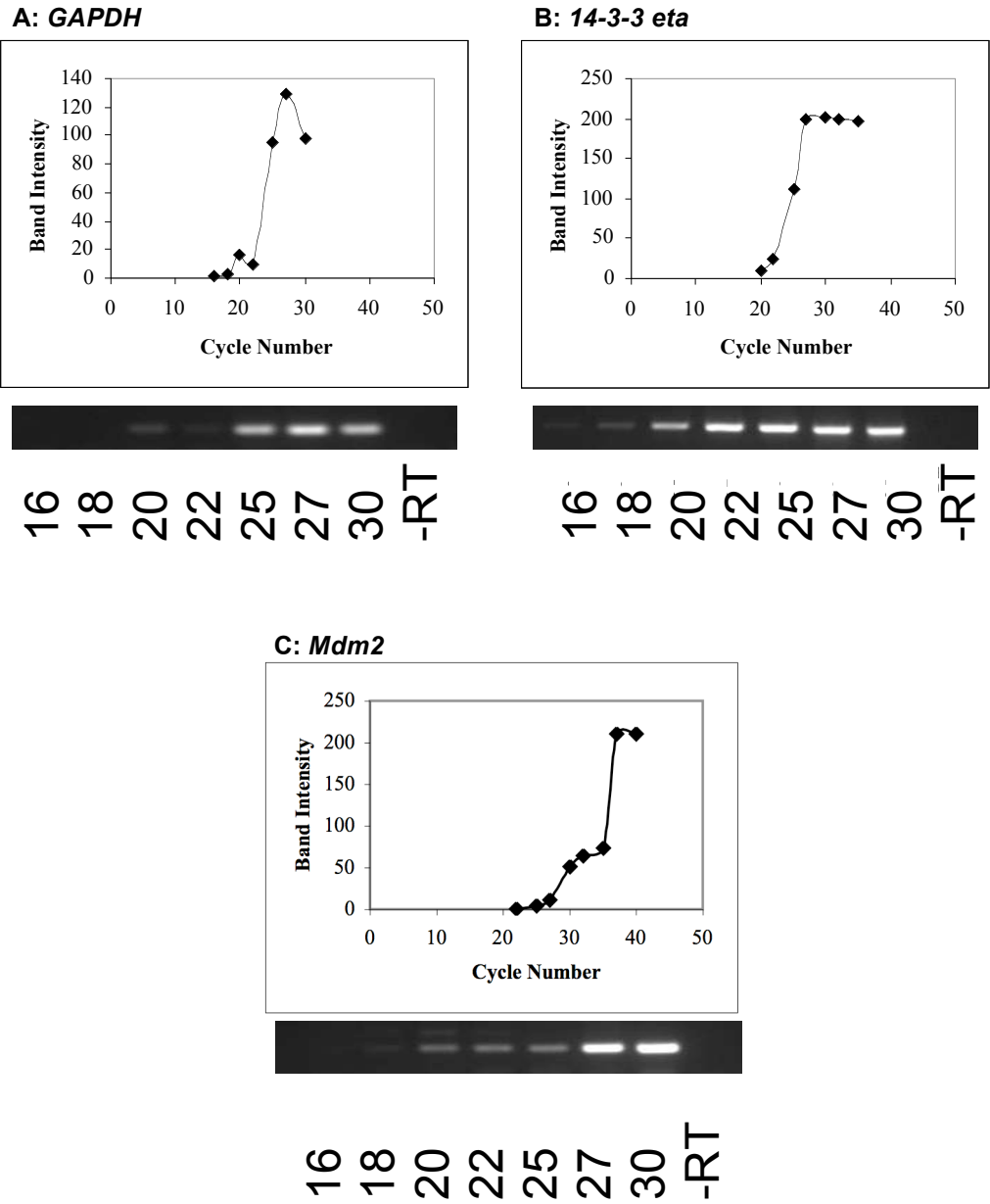

Supplement: Supplementary Fig. 3 — Standard curves for semi-quantitative PCR of chicken embryo lens. Standard curves were completed for three genes expressed at relatively high (GAPDH), intermediate (13-3-3 eta) and low levels (Mdm2) to determine the number of cycles to be used to ensure the PCR was in the linear phase of the reaction. The increase in band intensity with increasing cycles from 16–30 (shown underneath each graph) was used to produce the curve. –RT: no RT control. From these results, the cycle number used for each of these primer pairs was determined: GAPDH: 22 cycles; 14-3-3 eta: 25 cycles; Mdm2: 30 cycles. [file mmc3.pdf]

**A**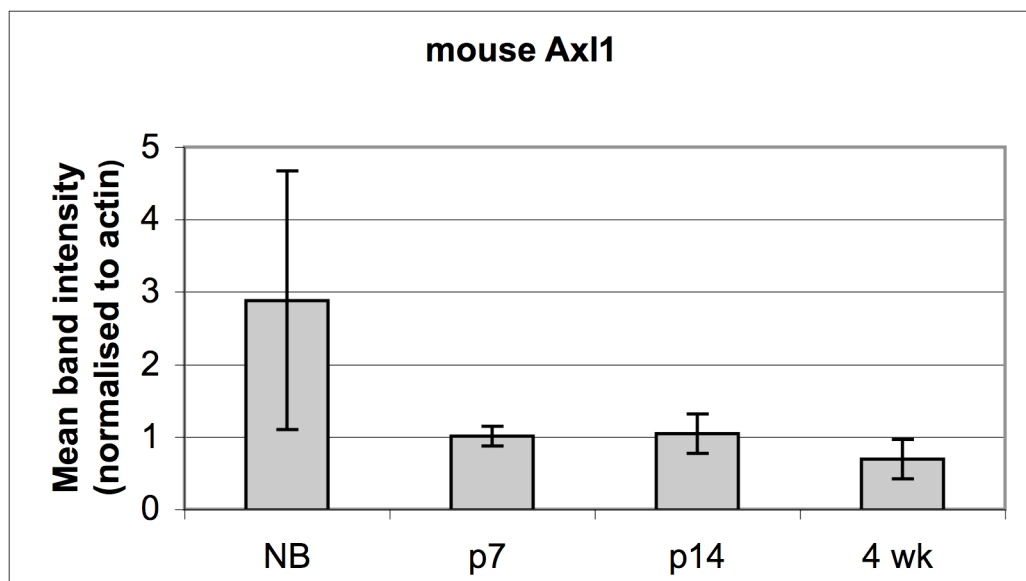**B**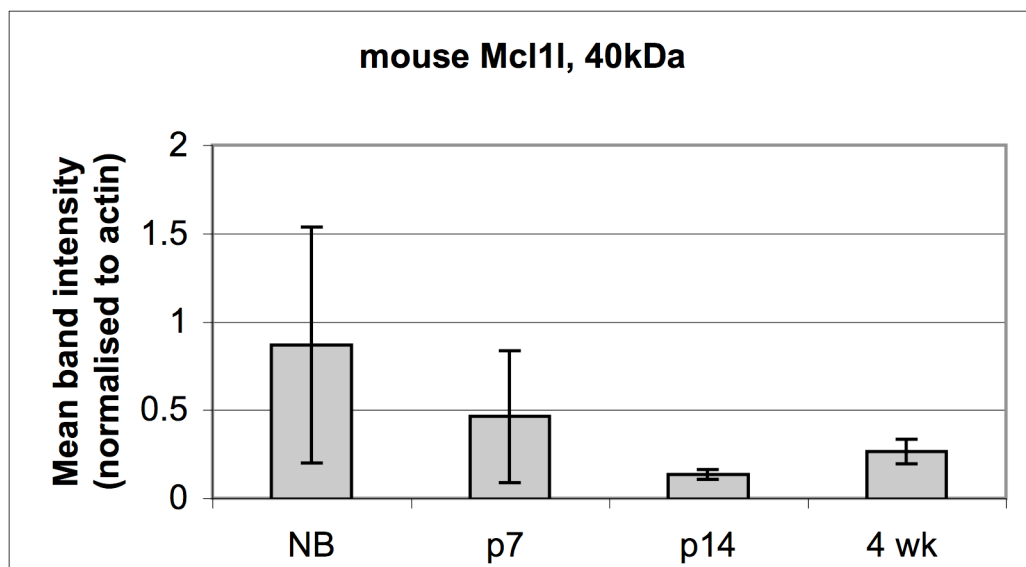**C**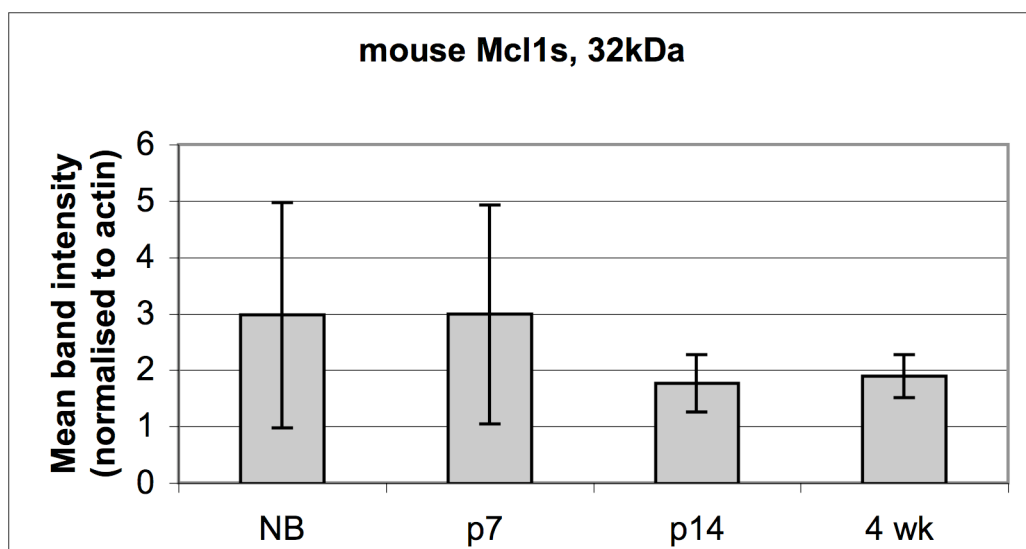

Supplement: Supplementary Fig. 4 — Graphical representations of representative Western blots using densitometry normalised against the actin control at each stage examined for mouse Axl-1 and Mcl-1. Error bars represent standard deviation of the mean for a minimum of 3 replicates in each case. (A) Axl expression peaked at Nb and had a constant low level of expression thereafter to 4 wk. (B) The short (32 kDa; pro-apoptotic) form of the blc-2 family member Mcl-1S had highest expression at Nb and p7, with lower expression at p14 and 4 wk, while expression of the long (40 kDa; anti-apoptotic) Mcl-1L was low at all stages examined with marginally higher expression at Nb. [file mmc4.pdf]

### Supplementary Figure 3: Geatrell et al (2008)

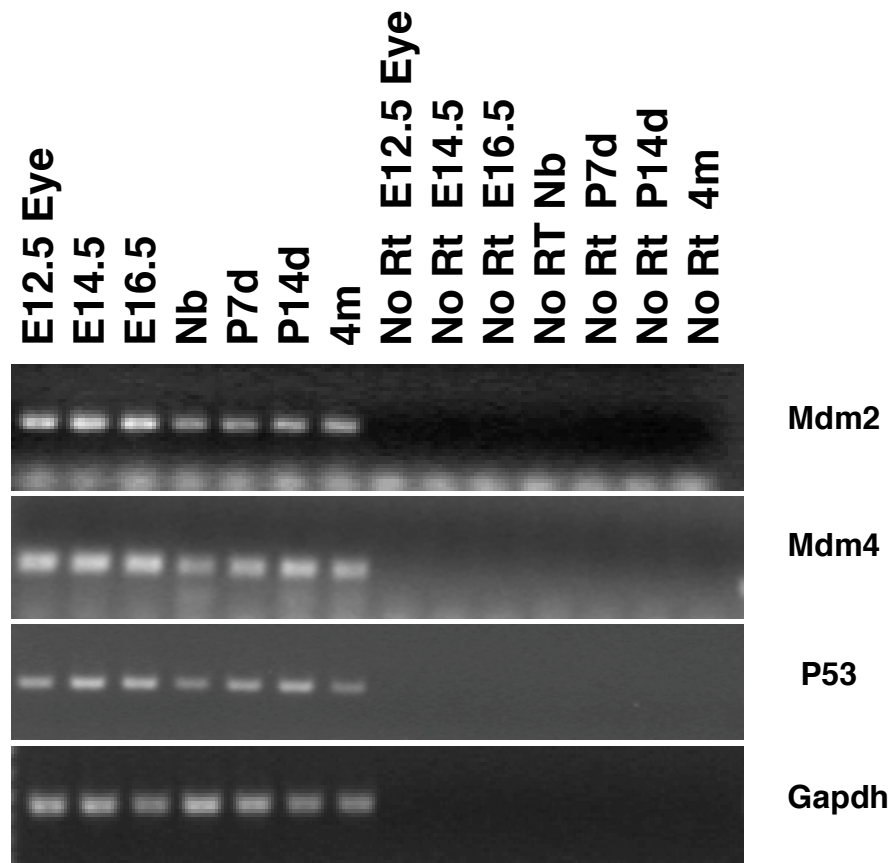

Supplement: Supplementary Fig. 5 — RT-PCRs reveal expression of Mdm2, Mdm4/X and p53 during embryonic stages of mouse lens development. PCRs were also carried out on cDNA prepared as described in Materials and Methods from E12.5 pooled mouse embryo whole eyes and on pooled lenses from E14.5, E16.5 as well as the post-natal stages examined previously. All three genes were differentially regulated at relatively high levels, compared to post-natal stages in the embryonic eyes/lenses. We were unable to obtain sufficient protein lysate for Western blotting from these stages of lens development. [file mmc5.pdf]

**A**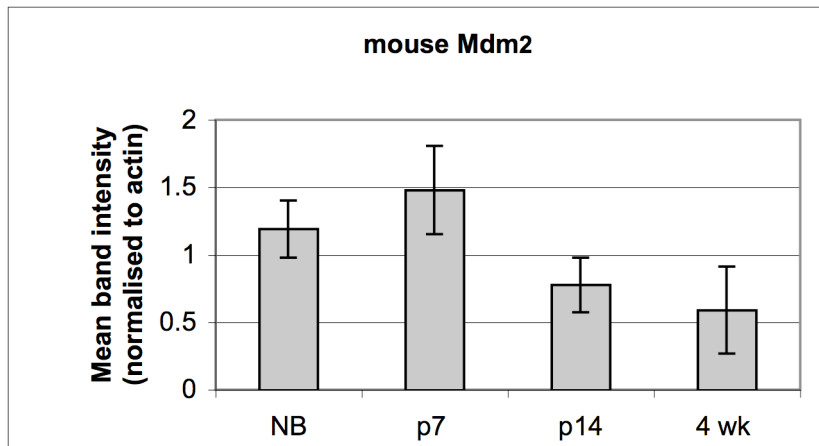**B**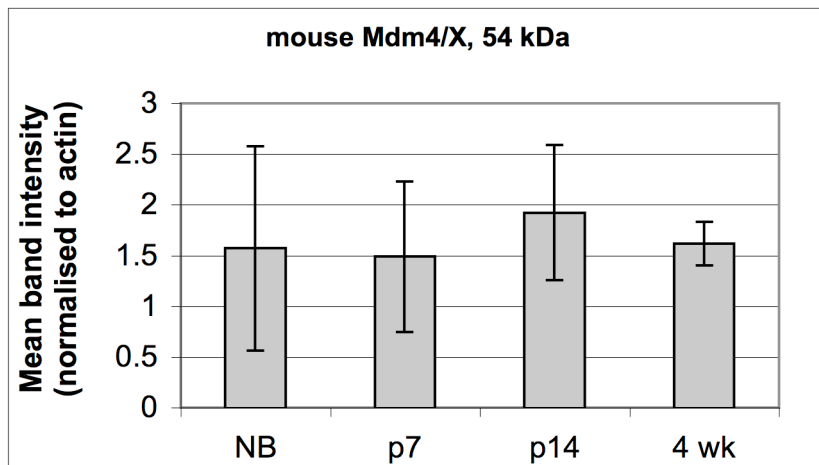**C**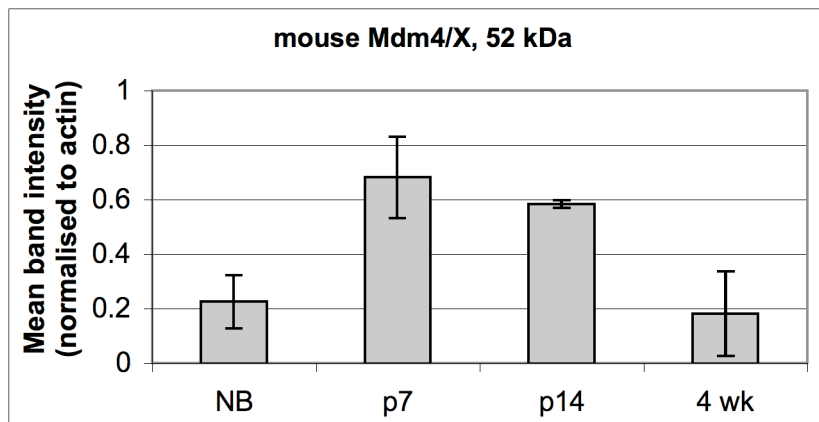**D**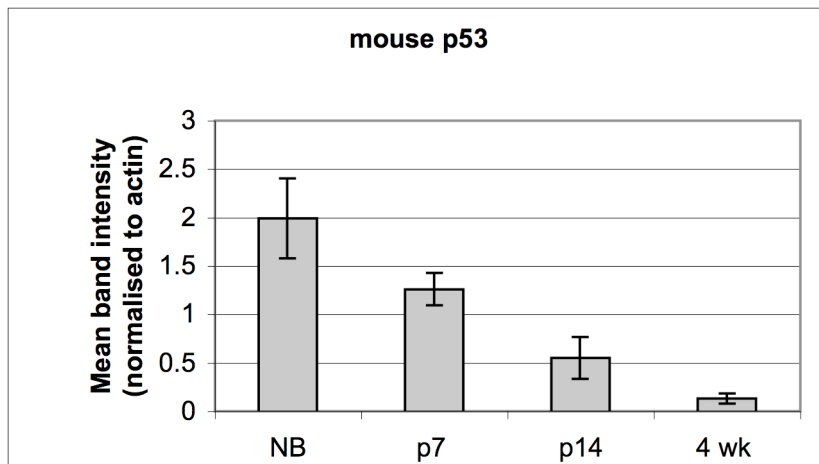

Supplement: Supplementary Fig. 6 — Graphical representations of representative Western blots using densitometry normalised against the actin control at each stage examined for mouse Mdm2, Mdm4/X and p53. Error bars represent standard deviation of the mean for a minimum of 3 replicates in each case. (A) Mdm2 expression at approximately 55 kDa was detected in the Nb lens, peaked at p7 and was reduced at p14 to 4 wk. (B and C) Mdm4/X was expressed as a doublet with a higher (more intense) band at approximately 54 kDa (B) and a lower (fainter) band at approximately 52 kDa. The band at 54 kDa band had a relatively constant level of expression across all stages examined, while the lower band of the doublet was expressed at lower levels with a peak of expression at p7-p14. (D) Expression of p53 peaked at Nb becoming virtually undetectable at 4 wk. [file mmc6.pdf]

**A**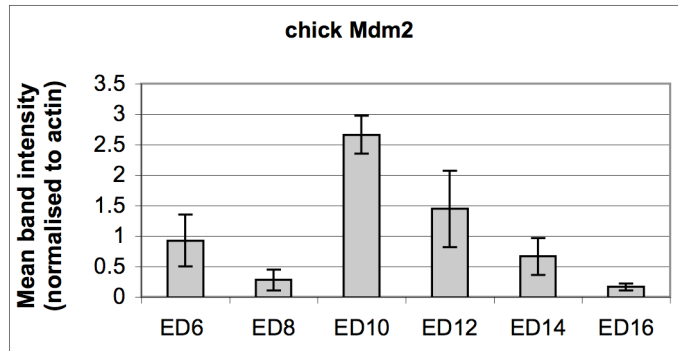**B**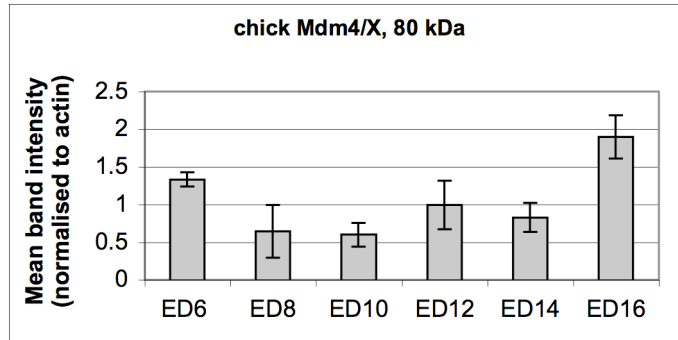**C**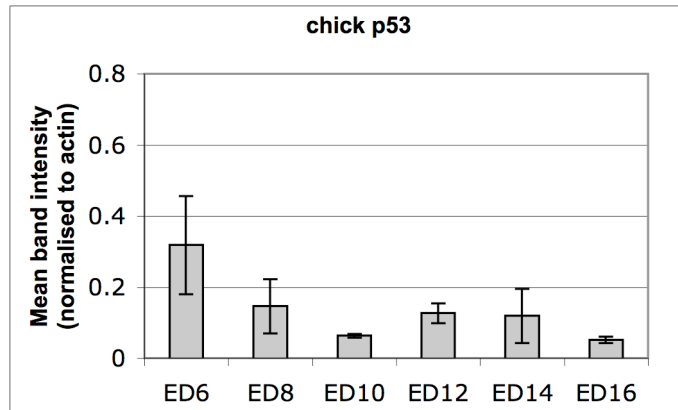**D**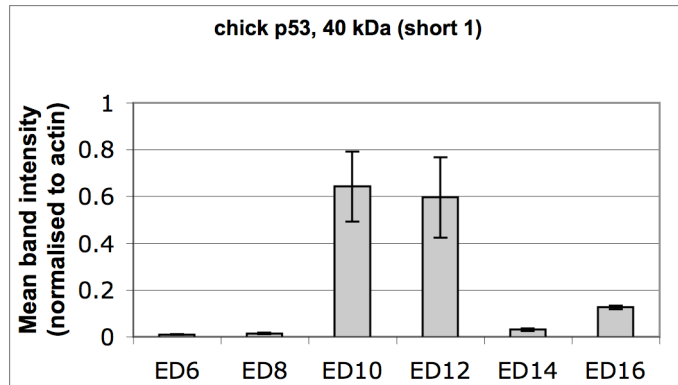**E**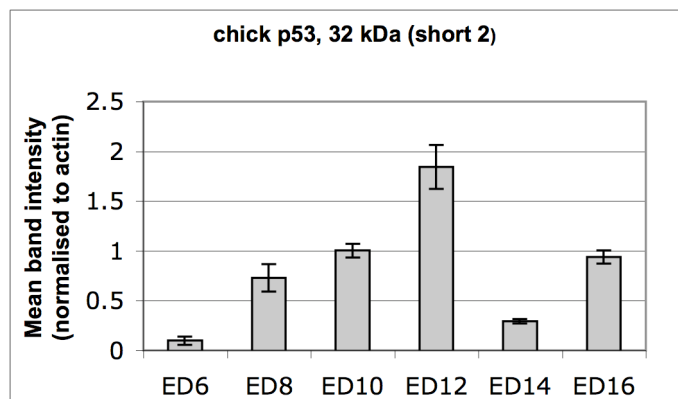

Supplement: Supplementary Fig. 7 — Graphical representations of representative Western blots using densitometry normalised against the actin control at each stage examined for chick Mdm2, Mdm4/X and p53. Error bars represent standard deviation of the mean for a minimum of 3 replicates in each case. (A) The Mdm2-positive band at 55 kDa was faint at embryonic day (ED)6-ED8, peaked in intensity at ED10-ED12 and was reduced in expression from ED14 to ED16. (B) Mdm4/X at 80 kDa showed a level of expression at all stages with highest expression at ED16. (C) Expression of p53 at 53 kDa in the chick lens samples was low at all stages, but expression was marginally higher at ED6. (D) Expression of a 40 kDa p53-positive band peaked at ED10-ED12. (E) Expression of a 32 kDa p53-positive band peaked at ED10-ED12 was reduced at ED14 and increased again at ED16. [file mmc7.pdf]
