# Supplementary material for: Apoptosis gene profiling reveals spatio-temporal regulated expression of the p53/Mdm2 pathway during lens development
Source: Exp Eye Res. 2009 Jun 1;88(6):1137–51. doi: 10.1016/j.exer.2009.01.020 (PMC2706329; doi:10.1016/j.exer.2009.01.020)
Supplement: Supplementary file 8 [file mmc8.pdf]

Supplementary Table 1: Details of all genes printed on the Panorama mouse apoptosis arrays (Sigma-Genosys, UK) used in these studies.

Array Coordinate	Gene Family	Gene Name	Accession Number	Gene Description
A1	Genomic DNA	GEN	N/A	Genomic DNA
P1	Genomic DNA	GEN	N/A	Genomic DNA
B2	Apoptosis-Related Factors	ABP1	AA109909	mp10d09.r1 Life Tech mouse embryo 8 5dpc 10664019 Mus musculus cDNA clone IMAGE:568817 5' similar to gb:U11863 AMILORIDE-SENSITIVE AMINE OXIDASE (HUMAN);, mRNA sequence
C2	Apoptosis-Related Factors	ADAM17	NM_009615	Mus musculus a disintegrin and metalloproteinase domain 17 (Adam17), mRNA
D2	Apoptosis-Related Factors	API5	NM_007466	Mus musculus apoptosis inhibitor 5 (Api5), mRNA
E2	Apoptosis-Related Factors	ATM	NM_007499	Mus musculus ataxia telangiectasia gene mutated in human beings (Atm), mRNA
F2	Apoptosis-Related Factors	CAD	NM_007859	Mus musculus DNase inhibited by DNA fragmentation factor (Didff), mRNA
G2	Apoptosis-Related Factors	CAV2	AF141322	Mus musculus caveolin-2 mRNA, complete cds
H2	Apoptosis-Related Factors	CD47	NM_010581	Mus musculus integrin-associated protein (Itgp), mRNA
I2	Apoptosis-Related Factors	CHML	AF189156	Mus musculus rab escort protein-2 (Chml) gene, complete cds
J2	Apoptosis-Related Factors	CIDE-A	NM_007702	Mus musculus cell death-inducing DNA fragmentation factor, alpha subunit-like effector A (Cidea), mRNA
K2	Apoptosis-Related Factors	CLDN3	NM_009902	Mus musculus claudin 3 (Cldn3), mRNA
L2	Apoptosis-Related Factors	Cln3	NM_009907	Mus musculus ceroid lipofuscinosis, neuronal 3, juvenile (Batten, Spielmeyer-Vogt disease) (Cln3), mRNA
M2	Apoptosis-Related Factors	Clu	NM_013492	Mus musculus alpha-clustrin and beta-clustrin mRNA, complete cds
N2	Apoptosis-Related Factors	Cox-1/Ptgs1	NM_008969	Mus musculus prostaglandin-endoperoxide synthase 1 (Ptgs1), mRNA
O2	Apoptosis-Related Factors	Cox-2/Ptgs2	NM_011198	Mus musculus prostaglandin-endoperoxide synthase 2 (Ptgs2), mRNA
B3	Apoptosis-Related Factors	CAS/CSE1	AI549625	ve53d06.y1 Beddington mouse embryonic region Mus musculus cDNA clone IMAGE:821867 5' similar to SW:CAS_HUMAN P55060 CELLULAR APOPTOSIS SUSCEPTIBILITY PROTEIN. ;, mRNA sequence
C3	Apoptosis-Related Factors	Ctsd	NM_009983	Mus musculus cathepsin D (Ctsd), mRNA
D3	Apoptosis-Related Factors	Cytochrome p450 oxidoreductase	NM_008898	Mus musculus P450 (cytochrome) oxidoreductase (Por), mRNA
E3	Apoptosis-Related Factors	DAD-1	NM_010015	Mus musculus defender against cell death 1 (Dad1), mRNA

Array Coordinate	Gene Family	Gene Name	Accession Number	Gene Description
F3	Apoptosis-Related Factors	Dap1	AI196645	ui53d07.y1 Sugano mouse liver mlia Mus musculus cDNA clone IMAGE:1886125 5' similar to SW:DAP1_HUMAN P51397 DEATH-ASSOCIATED PROTEIN 1 ;, mRNA sequence
G3	Apoptosis-Related Factors	DAXX	NM_007829	Mus musculus Fas death domain-associated protein (Daxx), mRNA
H3	Apoptosis-Related Factors	DEDD	NM_011615	Mus musculus tumor necrosis factor (ligand) superfamily, member 19 (Tnfsf19-pending), mRNA
I3	Apoptosis-Related Factors	DNAse1	NM_010061	Mus musculus mRNA for deoxyribonuclease I, complete cds
J3	Apoptosis-Related Factors	DNAse2	NM_010062	Mus musculus deoxyribonuclease II (Dnase2), mRNA
K3	Apoptosis-Related Factors	Fem1B	NM_010193	Mus musculus feminization 1 b homolog (C. elegans) (Fem1b), mRNA
L3	Apoptosis-Related Factors	FLASH	NM_011997	Mus musculus caspase 8 associated protein 2 (Casp8ap2), mRNA
M3	Apoptosis-Related Factors	Galectin-3	X16834	Mouse mRNA for Mac-2 antigen
N3	Apoptosis-Related Factors	GAPDH	NM_008084	Mus musculus glyceraldehyde-3-phosphate dehydrogenase (Gapd), mRNA
O3	Apoptosis-Related Factors	GPX1	NM_008160	Mus musculus glutathione peroxidase 1 (Gpx1), mRNA
B4	Apoptosis-Related Factors	GSN	NM_010354	Mus musculus gelsolin (Gsn), mRNA
C4	Apoptosis-Related Factors	HD	U24233	Mus musculus huntingtin (Hd) mRNA, complete cds
D4	Apoptosis-Related Factors	HnrpA1	NM_010447	Mus musculus heterogeneous nuclear ribonucleoprotein A1 (Hnrpa1), mRNA
E4	Apoptosis-Related Factors	ICAD/DFFA	NM_010044	Mus musculus DNA fragmentation factor, alpha subunit (Dffa), mRNA
F4	Apoptosis-Related Factors	Integrin-αV	NM_008402	Mus musculus integrin alpha V (Cd51) (Itgav), mRNA
G4	Apoptosis-Related Factors	MFGE8	NM_008594	Mus musculus milk fat globule-EGF factor 8 protein (Mfge8), mRNA
H4	Apoptosis-Related Factors	PGES	AB041997	Mus musculus PGES mRNA for prostaglandin E synthase, complete cds.
I4	Apoptosis-Related Factors	MT-2	K02236	vf04g05.y1 Knowles Solter mouse blastocyst B3 Mus musculus cDNA clone IMAGE:834776 5' similar to gb:K02236 Mouse metallothionein II (MOUSE);, mRNA sequence
J4	Apoptosis-Related Factors	eNOS	NM_008713	Mus musculus nitric oxide synthase 3, endothelial cell (Nos3), mRNA
K4	Apoptosis-Related Factors	iNOS	NM_010927	Mus musculus nitric oxide synthase 2, inducible, macrophage (Nos2), mRNA
L4	Apoptosis-Related Factors	nNOS	NM_008712	Mus musculus nitric oxide synthase 1, neuronal (Nos1), mRNA
M4	Apoptosis-Related Factors	ODC	NM_013614	Mus musculus ornithine decarboxylase, structural (Odc), mRNA
N4	Apoptosis-Related Factors	P2RX1	NM_008771	Mus musculus purinergic receptor P2X, ligand-gated ion channel, 1 (P2rx1), mRNA
O4	Apoptosis-Related Factors	uPAR1	NM_011113	Mus musculus urokinase plasminogen activator receptor (Plaur), mRNA

Array Coordinate	Gene Family	Gene Name	Accession Number	Gene Description
B5	Apoptosis-Related Factors	PDCD1	NM_008798	Mus musculus programmed cell death 1 (Pdcd1), mRNA
C5	Apoptosis-Related Factors	PDCD2	NM_008799	Mus musculus programmed cell death 2 (Pdcd2), mRNA
D5	Apoptosis-Related Factors	PIGA	D26047	Mouse testis mRNA for Pig-a
E5	Apoptosis-Related Factors	PIN	AF020185	mj05g09.r1 Soares mouse embryo NbME13.5 14.5 Mus musculus cDNA clone IMAGE:475264 5' similar to WP:T26A5.9 CE00788 ;, mRNA sequence
F5	Apoptosis-Related Factors	PLA2G1B	NM_011107	Mus musculus phospholipase A2, group IB, pancreas (Pla2g1b), mRNA
G5	Apoptosis-Related Factors	ZAC1/PLAGL1	X95504	M.musculus mRNA for zinc finger protein
H5	Apoptosis-Related Factors	PRKR	NM_011163	Mus musculus eukaryotic translation initiation factor 2 alpha kinase 2 (Eif2ak2), mRNA
I5	Apoptosis-Related Factors	REQ	NM_011262	Mus musculus requiem (Req), mRNA
J5	Apoptosis-Related Factors	RP105/Ly78	NM_008533	Mus musculus lymphocyte antigen 78 (Ly78), mRNA
K5	Apoptosis-Related Factors	Mts-1	X16190	Mouse mts1 gene
L5	Apoptosis-Related Factors	SAG-1	AF092877	Mus musculus zinc RING finger protein SAG mRNA, complete cds
M5	Apoptosis-Related Factors	SARP-1	NM_009144	Mus musculus stromal cell derived factor 5 (Sdf5), mRNA
N5	Apoptosis-Related Factors	SARP-2/sFRP-1	U88566	Mus musculus secreted frizzled related protein sFRP-1 (Sfrp1) mRNA, complete cds
O5	Apoptosis-Related Factors	sFRP-5/SARP-3	AF117759	Mus musculus secreted frizzled-related protein 5 (Sfrp5) mRNA, complete cds
B6	Apoptosis-Related Factors	SIAH1	NM_009172	Mus musculus seven in absentia 1A (Siah1a), mRNA
C6	Apoptosis-Related Factors	SREBF1	AB017337	Mus musculus mRNA for sterol regulatory element-binding protein-1 (SREBP-1), partial cds
D6	Apoptosis-Related Factors	SREBF2	AW106709	um32e12.y1 Sugano mouse kidney mkia Mus musculus cDNA clone IMAGE:2236270 5' similar to SW:SRE2_CRIGR Q60429 STEROL REGULATORY ELEMENT BINDING PROTEIN-2 ;, mRNA sequence
E6	Apoptosis-Related Factors	TDAG8	NM_008152	Mus musculus G-protein coupled receptor 25 (Gpcr25), mRNA
F6	Apoptosis-Related Factors	TFAR15	AF159368	Mus musculus TF-1 cell apoptosis related protein-15 (Tfar15) mRNA, complete cds
G6	Apoptosis-Related Factors	Thrombospondin	NM_011580	Mouse thrombospondin 1 mRNA, complete cds
H6	Apoptosis-Related Factors	TIAF1	AF104984	Mus musculus TGF-b1-induced anti-apoptotic factor 1 mRNA, complete cds
I6	Apoptosis-Related Factors	TIAL1	NM_009383	Mus musculus Tial1 cytotoxic granule-associated RNA-binding protein-like 1 (Tial1), mRNA
J6	Apoptosis-Related Factors	TSSC3	NM_009434	Mus musculus tumor-suppressing subchromosomal transferable fragment 3 (Tssc3), mRNA

Array Coordinate	Gene Family	Gene Name	Accession Number	Gene Description
K6	Apoptosis-Related Factors	TXN	NM_011660	Mus musculus thioredoxin (Txn), mRNA
L6	Caspases and Regulators	Caspase-1	NM_009807	Mus musculus caspase 1 (Casp1), mRNA
M6	Caspases and Regulators	Caspase-2	NM_007610	Mus musculus caspase 2 (Casp2), mRNA
N6	Caspases and Regulators	Caspase-3	NM_009810	Mus musculus caspase 3, apoptosis related cysteine protease (Casp3), mRNA
O6	Caspases and Regulators	Caspase-6	NM_009811	Mus musculus caspase 6 (Casp6), mRNA
B7	Caspases and Regulators	Caspase-7	NM_007611	Mus musculus caspase 7 (Casp7), mRNA
C7	Caspases and Regulators	Caspase-8	AJ007749	Mus musculus mRNA for caspase-8
D7	Caspases and Regulators	Caspase-9	NM_015733	Mus musculus mRNA for caspase9, complete cds
E7	Caspases and Regulators	Caspase-11	NM_007609	Mus musculus caspase 11 (Casp11), mRNA
F7	Caspases and Regulators	Caspase-12	NM_009808	Mus musculus caspase 12 (Casp12), mRNA
G7	Caspases and Regulators	Caspase-14	NM_009809	Mus musculus caspase 14 (Casp14), mRNA
H7	Caspases and Regulators	FLIPL/Cash	NM_009805	Mus musculus caspase homolog (Cash), mRNA
I7	Caspases and Regulators	Granzyme B	NM_013542	Mus musculus granzyme B (Gzmb), mRNA
J7	Caspases and Regulators	Granzyme A	NM_010370	Mus musculus granzyme A (Gzma), mRNA
K7	Caspases and Regulators	cIAP-1	NM_007464	Mus musculus apoptosis inhibitor 1 (Api1), mRNA
L7	Caspases and Regulators	cIAP-2	NM_007465	Mus musculus apoptosis inhibitor 2 (Api2), mRNA
M7	Caspases and Regulators	NAIP	NM_010872	Mus musculus neuronal apoptosis inhibitory protein 2 (Naip2), mRNA
N7	Caspases and Regulators	PARP	NM_007415	Mus musculus ADP-ribosyltransferase (NAD+; poly (ADP-ribose) polymerase) 1 (Adprt1), mRNA
O7	Caspases and Regulators	PARP-2	NM_009632	Mus musculus ADP-ribosyltransferase (NAD+; poly (ADP-ribose) polymerase) 2 (Adprt2), mRNA
B8	Caspases and Regulators	Sentrin/UBL1	NM_009460	Mus musculus ubiquitin-like 1 (Ubl1), mRNA
C8	Caspases and Regulators	Survivin	NM_009689	Mus musculus apoptosis inhibitor 4 (Api4), mRNA
D8	Caspases and Regulators	XIAP	NM_009688	Mus musculus apoptosis inhibitor 3 (Api3), mRNA
E8	Cell Cycle Regulators	53BP2	U58881	Mus musculus p53 binding protein 2 homolog (p53BP2), partial cDNA
F8	Cell Cycle Regulators	APEX/Ref-1	NM_009687	Mus musculus apurinic/apyrimidinic endonuclease (Apex), mRNA
G8	Cell Cycle Regulators	Calcyclin	NM_011313	Mus musculus calcium binding protein A6 (calcyclin) (S100a6), mRNA
H8	Cell Cycle Regulators	CBP	S66385	CREB-binding protein [mice, brain, mRNA Partial, 7326 nt]

Array Coordinate	Gene Family	Gene Name	Accession Number	Gene Description
I8	Cell Cycle Regulators	CDC2	NM_007659	Mus musculus cell division cycle 2 homolog A (S. pombe) (Cdc2a), mRNA
J8	Cell Cycle Regulators	CDK2	NM_016756	Mus musculus mRNA for cyclin dependent kinase (CDK2L) - the 39 kDa variant of CDK2
K8	Cell Cycle Regulators	CDK4	NM_009870	Mus musculus cyclin-dependent kinase 4 (Cdk4), mRNA
L8	Cell Cycle Regulators	CDK5	NM_007668	Mus musculus cyclin-dependent kinase 5 (Cdk5), mRNA
M8	Cell Cycle Regulators	CDK6	AF132483	Mus musculus strain BALB/c cyclin-dependent kinase 6 (Cdk6) mRNA, complete cds
N8	Cell Cycle Regulators	Cyclin A	NM_009828	Mus musculus cyclin A2 (Ccna2), mRNA
O8	Cell Cycle Regulators	Cyclin D1	M64403	Mus domesticus cyclin-like protein (induced by colony-stimulating factor 1) (CYL-1) mRNA, complete cds
B9	Cell Cycle Regulators	Cyclin G1	NM_009831	Mus musculus cyclin G (Ccng), mRNA
C9	Cell Cycle Regulators	DP1	NM_009361	Mus musculus transcription factor Dp 1 (Tfdp1), mRNA
D9	Cell Cycle Regulators	MDM2	NM_010786	Mus musculus transformed mouse 3T3 cell double minute 2 (Mdm2), mRNA
E9	Cell Cycle Regulators	c-myc	NM_010849	Mus musculus myelocytomatosis oncogene (Myc), mRNA
F9	Cell Cycle Regulators	p15INK4b/CDKN2B	NM_007670	Mus musculus cyclin-dependent kinase inhibitor 2B (p15, inhibits CDK4) (Cdkn2b), mRNA
G9	Cell Cycle Regulators	p19/NSG2	NM_008741	Mus musculus neuron specific gene family member 2 (Nsg2), mRNA
H9	Cell Cycle Regulators	RBL1/p107	U27177	Mus musculus p107 (p107) mRNA, complete cds
I9	Cell Cycle Regulators	RBL2/p130	NM_011250	Mus musculus retinoblastoma-like 2 (Rbl2), mRNA
J9	Cell Cycle Regulators	P300	AA881090	vz06c10.r1 Soares_mammary_gland_NbMMG Mus musculus cDNA clone IMAGE:1314930 5' similar to TR:Q09472 Q09472 E1A-ASSOCIATED PROTEIN P300. ;, mRNA sequence
K9	Cell Cycle Regulators	PAK1	NM_011035	Mus musculus p21 (CDKN1A)-activated kinase 1 (Pak1), mRNA
L9	Cell Cycle Regulators	PCNA	NM_011045	Mus musculus proliferating cell nuclear antigen (Pcna), mRNA.
M9	Cell Cycle Regulators	pRB	NM_009029	Mus musculus retinoblastoma 1 (Rb1), mRNA
N9	Cell Cycle Regulators	RBBP4/RbAp48	NM_009030	Mus musculus retinoblastoma binding protein 4 (Rbbp4), mRNA
O9	Cell Cycle Regulators	RBP1	AA474808	ve62d06.r1 Beddington mouse embryonic region Mus musculus cDNA clone IMAGE:822731 5' similar to gb:S66427 RETINOBLASTOMA BINDING PROTEIN 1 (HUMAN);, mRNA sequence
B10	Cell Cycle Regulators	RBBP6/PACT/RBQ1	U28789	Mus musculus p53-associated cellular protein PACT mRNA, partial cds
C10	Cell Cycle Regulators	Sp1	NM_013672	Mus musculus transcription factor Sp1 mRNA, complete cds
D10	Cell Cycle Regulators	TRP53/p53	NM_011640	Mus musculus transformation related protein 53 (Trp53), mRNA

Array Coordinate	Gene Family	Gene Name	Accession Number	Gene Description
E10	Cytokines and Receptors	AR	NM_013476	Mouse mRNA for Tfm androgen receptor (inactive)
F10	Cytokines and Receptors	ART/Agrp	NM_007427	Mus musculus agouti related protein (Agrp), mRNA
G10	Cytokines and Receptors	Axl	NM_009465	Mus musculus AXL receptor tyrosine kinase (Axl), mRNA
H10	Cytokines and Receptors	Dtk/Tyro3	U18933	Mus musculus receptor tyrosine kinase (Dtk) mRNA, complete cds
I10	Cytokines and Receptors	EGF	J00380	mouse epidermal growth factor (egf) mrna
J10	Cytokines and Receptors	EGF R	NM_007912	Mus musculus epidermal growth factor receptor (Egfr), mRNA
K10	Cytokines and Receptors	erbB2	U71126	Mus musculus erbB2 mRNA, partial cds
L10	Cytokines and Receptors	erbB3	L47240	Mus musculus tyrosine kinase (erbB3) mRNA, partial cds
M10	Cytokines and Receptors	erbB4	AF059176	Mus musculus EGF-like growth factor receptor ErbB4 extracellular domain mRNA, partial cds
N10	Cytokines and Receptors	GAS1	NM_008086	Mus musculus growth arrest specific 1 (Gas1), mRNA
O10	Cytokines and Receptors	GAS2	NM_008087	Mus musculus growth arrest specific 2 (Gas2), mRNA
B11	Cytokines and Receptors	GM-CSF	X02333	Murine mRNA for granulocyte-macrophage colony stimulating factor (GM-GSF)
C11	Cytokines and Receptors	GM-CSF Rα	NM_009970	Mus musculus colony stimulating factor 2 receptor, alpha, low-affinity (granulocyte-macrophage) (Csf2ra), mRNA
D11	Cytokines and Receptors	IFN-γ R1	NM_010511	Mus musculus interferon gamma receptor (Ifngr), mRNA
E11	Cytokines and Receptors	IFN-γ R2	NM_008338	Mus musculus interferon gamma receptor 2 (Ifngr2), mRNA
F11	Cytokines and Receptors	IGF-I	NM_010512	Mus musculus insulin-like growth factor 1 (IGF1), mRNA
G11	Cytokines and Receptors	IGF-II	NM_010514	Mus musculus insulin-like growth factor 2 (IGF2), mRNA
H11	Cytokines and Receptors	IGF R	AF056187	Mus musculus insulin-like growth factor I receptor mRNA, complete cds
I11	Cytokines and Receptors	IL-1 R AcP	NM_008364	Mus musculus interleukin 1 receptor accessory protein (Il1rap), mRNA
J11	Cytokines and Receptors	IL-1ra	M57525	Mouse interleukin 1 receptor antagonist (IL1RA) mRNA, complete cds
K11	Cytokines and Receptors	IL-1 RI	NM_008362	Mus musculus interleukin 1 receptor, type I (Il1r1), mRNA
L11	Cytokines and Receptors	IL-1 RII	NM_010555	Mus musculus interleukin 1 receptor, type II (Il1r2), mRNA
M11	Cytokines and Receptors	IL-1α	NM_010554	Mus musculus interleukin 1 alpha (Il1a), mRNA
N11	Cytokines and Receptors	IL-1β	NM_008361	Mus musculus interleukin 1 beta (Il1b), mRNA
O11	Cytokines and Receptors	IL-2	NM_008366	Mouse mRNA for interleukin-2 (IL-2)
B12	Cytokines and Receptors	IL-2 Rα	NM_008367	Mus musculus interleukin 2 receptor, alpha chain (Il2ra), mRNA

Array Coordinate	Gene Family	Gene Name	Accession Number	Gene Description
C12	Cytokines and Receptors	IL-2 Rβ	NM_008368	Mus musculus interleukin 2 receptor, beta chain (Il2rb), mRNA
D12	Cytokines and Receptors	IL-2 Rγ	NM_013563	Mouse interleukin 2 receptor gamma chain mRNA, complete cds
E12	Cytokines and Receptors	IL-4	M25892	Mus musculus interleukin 4 (Il-4) mRNA, complete cds
F12	Cytokines and Receptors	IL-4 Rα	NM_010557	Mus musculus interleukin 4 receptor, alpha (Il4ra), mRNA
G12	Cytokines and Receptors	IL-10	NM_010548	Mus musculus interleukin 10 (Il10), mRNA
H12	Cytokines and Receptors	IL-10 Rα	NM_008348	Mus musculus interleukin 10 receptor, alpha (Il10ra), mRNA
I12	Cytokines and Receptors	IL-12 p35	M86672	Mus musculus interleukin 12 p35 subunit, complete cds
J12	Cytokines and Receptors	IL-12 p40	M86671	Mus musculus interleukin 12 p40 subunit, complete cds
K12	Cytokines and Receptors	IL-12 Rβ1	NM_008353	Mus musculus interleukin 12 receptor, beta 1 (Il12rb1), mRNA
L12	Cytokines and Receptors	IL-12 Rβ2	NM_008354	Mus musculus interleukin 12 receptor, beta 2 (Il12rb2), mRNA
M12	Cytokines and Receptors	IL-13	NM_008355	Mus musculus interleukin 13 (Il13), mRNA
N12	Cytokines and Receptors	IL-13 Rα1	S80963	NR4=IL-13 receptor alpha chain [mice, embryonal stem cell, Genomic/mRNA, 1680 nt]
O12	Cytokines and Receptors	IL-15	NM_008357	Mus musculus interleukin 15 (Il15), mRNA
B13	Cytokines and Receptors	IL-15 Rα	NM_008358	Mus musculus interleukin 15 receptor, alpha chain (Il15ra), mRNA
C13	Cytokines and Receptors	Mannose 6-phosphate R	U04710	Mus musculus domesticus C57 Black 6 x CBA cation-independent mannose 6-phosphate/insulin-like growth factor II receptor precursor mRNA, complete cds
D13	Cytokines and Receptors	M-CSF	NM_007778	Mus musculus colony stimulating factor 1 (macrophage) (Csf1), mRNA
E13	Cytokines and Receptors	M-CSF R	NM_007779	Mus musculus colony stimulating factor 1 receptor (Csf1r), mRNA
F13	Cytokines and Receptors	Prolactin	NM_011164	Mus musculus prolactin (Prl), mRNA
G13	Cytokines and Receptors	TGF-β	NM_011577	Mus musculus transforming growth factor, beta 1 (Tgfb1), mRNA
H13	Cytokines and Receptors	TGF-β2	NM_009367	Mus musculus transforming growth factor, beta 2 (Tgfb2), mRNA
I13	Cytokines and Receptors	TGF-β3	NM_009368	Mus musculus transforming growth factor, beta 3 (Tgfb3), mRNA
J13	Cytokines and Receptors	TGF-β RI	NM_007394	Mus musculus activin A receptor, type 1 (Acvr1), mRNA
K13	Cytokines and Receptors	TGF-β RII	NM_009371	Mus musculus transforming growth factor, beta receptor II (Tgfbr2), mRNA
L13	Cytokines and Receptors	TGF-β RIII	AF039601	Mus musculus betaglycan mRNA, complete cds
M13	Cytokines and Receptors	TrkB	NM_008745	Mus musculus neurotrophic tyrosine kinase, receptor, type 2 (Ntrk2), mRNA
N13	Cytokines and Receptors	TrkC	AF035400	Mus musculus neurotrophin-3 receptor non-catalytic isoform 2 (trkC) mRNA, complete cds

Array Coordinate	Gene Family	Gene Name	Accession Number	Gene Description
O13	Mitochondrial Associated	A1	L16462	Mus musculus hemopoietic-specific early response protein (A1) mRNA, complete cds
B14	Mitochondrial Associated	AIF/Pdcd8	NM_012019	Mus musculus programmed cell death 8 (apoptosis inducing factor) (Pdcd8), mRNA
C14	Mitochondrial Associated	Apaf1	NM_009684	Mus musculus apoptotic protease activating factor 1 (Apaf1), mRNA
D14	Mitochondrial Associated	Bad	NM_007522	Mus musculus Bcl-associated death promoter (Bad), mRNA
E14	Mitochondrial Associated	Bag-1	NM_009736	Mus musculus Bcl2-associated athanogene 1 (Bag1), mRNA
F14	Mitochondrial Associated	BAK	NM_007523	Mus musculus Bcl2 homologous antagonist/killer (Bak), mRNA
G14	Mitochondrial Associated	Bax-α	NM_007527	Mus musculus Bcl2-associated X protein (Bax), mRNA
H14	Mitochondrial Associated	Bcl-2	NM_009741	Mus musculus B-cell leukemia/lymphoma 2 (Bcl2), mRNA
I14	Mitochondrial Associated	Bcl-w	NM_007537	Mus musculus Bcl2-like 2 (Bcl2l2), mRNA
J14	Mitochondrial Associated	Bcl-x	NM_009743	Mus musculus Bcl2-like (Bcl2l), mRNA
K14	Mitochondrial Associated	BID	NM_007544	Mus musculus BH3 interacting domain death agonist (Bid), mRNA
L14	Mitochondrial Associated	Bim	NM_009754	Mus musculus Bcl2 interacting mediator of cell death (Bim), mRNA
M14	Mitochondrial Associated	Cytochrome C	NM_007808	Mus musculus cytochrome c, somatic (Cycs), mRNA
N14	Mitochondrial Associated	Mcl-1	NM_008562	Mus musculus myeloid cell leukaemia sequence 1 (Mcl1), mRNA
O14	Signal Transduction	14-3-3 eta	NM_011738	Mus musculus tyrosine 3-monooxygenase/tryptophan 5-monooxygenase activation protein, eta polypeptide (Ywhah), mRNA
B15	Signal Transduction	AKT/PKB	NM_009652	Mus musculus thymoma viral proto-oncogene (Akt), mRNA
C15	Signal Transduction	ALG4	AF055669	Mus musculus apoptosis-linked gene 4, F form (Alg-4) mRNA, partial cds
D15	Signal Transduction	ASK1/MAP3K5	NM_008580	Mus musculus mitogen activated protein kinase kinase kinase 5 (Map3k5), mRNA
E15	Signal Transduction	Bcl-10	NM_009740	Mus musculus B-cell leukemia/lymphoma 10 (Bcl10), mRNA
F15	Signal Transduction	CARDIAK	AA655189	vv13a12.r1 Stratagene mouse heart (#937316) Mus musculus cDNA clone IMAGE:1211518 5' similar to TR:G1236943 G1236943 RIP PROTEIN KINASE. ;, mRNA sequence
G15	Signal Transduction	CRADD	NM_009950	Mus musculus CASP2 and RIPK1 domain containing adaptor with death domain (Cradd), mRNA
H15	Signal Transduction	TRAF3/CRAF1	NM_011632	Mus musculus Tnf receptor-associated factor 3 (Traf3), mRNA
I15	Signal Transduction	DAP Kinase	AA620064	vl53d10.r1 Stratagene mouse skin (#937313) Mus musculus cDNA clone IMAGE:975955 5' similar to TR:G434847 G434847 DAP-KINASE. ;, mRNA sequence

Array Coordinate	Gene Family	Gene Name	Accession Number	Gene Description
J15	Signal Transduction	DRAK2	AI585389	vj56a01.y1 Knowles Solter mouse blastocyst B1 Mus musculus cDNA clone IMAGE:933000 5' similar to SW:DAPK_HUMAN P53355 DEATH-ASSOCIATED PROTEIN KINASE 1 ;, mRNA sequence
K15	Signal Transduction	E2F1	NM_007891	Mus musculus E2F transcription factor 1 (E2f1), mRNA
L15	Signal Transduction	FADD	NM_010175	Mus musculus Fas-associating protein with death domain (Fadd), mRNA
M15	Signal Transduction	FAN	NM_010945	Mus musculus neutral sphingomyelinase (N-SMase) activation associated factor (Nsmaf), mRNA
N15	Signal Transduction	GSK3B	AF156099	Mus musculus glycogen synthase kinase 3 beta mRNA, complete cds
O15	Signal Transduction	IKK-α	NM_007700	Mus musculus conserved helix-loop-helix ubiquitous kinase (Chuk), mRNA
B16	Signal Transduction	IKK-β	AF088910	Mus musculus IkB kinase-beta (Ikkb) mRNA, complete cds
C16	Signal Transduction	MADD	AI595199	mk16f10.y1 Soares mouse p3NMF19.5 Mus musculus cDNA clone IMAGE:493099 5' similar to TR:O08873 O08873 RAB3 GDP/GTP EXCHANGE PROTEIN. ;, mRNA sequence
D16	Signal Transduction	MEKK1	AF117340	Mus musculus MAP kinase kinase kinase 1 (Mekk1) mRNA, complete cds
E16	Signal Transduction	MYD118	NM_008655	Mus musculus myeloid differentiation primary response gene 118 (Myd118), mRNA
F16	Signal Transduction	NF-κB DNA binding subunit	NM_008689	Mus musculus nuclear factor of kappa light chain gene enhancer in B-cells 1, p105 (Nfkb1), mRNA
G16	Signal Transduction	NF-κB inducing kinase	NM_016896	Mus musculus Nfkb inducing kinase (Nik-pending), mRNA
H16	Signal Transduction	NFκBp65	NM_009045	Mus musculus avian reticuloendotheliosis viral (v-rel) oncogene homolog A (Rela), mRNA
I16	Signal Transduction	Par-4	AA023558	mh76e12.r1 Soares mouse placenta 4NbMP13.5 14.5 Mus musculus cDNA clone IMAGE:456910 5' similar to TR:G456282 G456282 CLONE PAR-4 INDUCED BY EFFECTORS OF APOPTOSIS. ;, mRNA sequence
J16	Signal Transduction	PI-3 Kinase	NM_011084	Mus musculus phosphatidylinositol 3-kinase, C2 domain containing, gamma polypeptide (Pik3c2g), mRNA
K16	Signal Transduction	PKC-α	NM_011101	Mus musculus protein kinase C, alpha (Pkca), mRNA
L16	Signal Transduction	PP2A	AI894369	mf46e11.y1 Soares mouse embryo NbME13.5 14.5 Mus musculus cDNA clone IMAGE:408140 5' similar to gb:M64929 PROTEIN PHOSPHATASE PP2A, 55 KD REGULATORY SUBUNIT, ALPHA (HUMAN);, mRNA sequence
M16	Signal Transduction	PTEN	NM_008960	Mus musculus phosphatase and tensin homolog (Pten), mRNA
N16	Signal Transduction	RARβ2	S56660	retinoic acid nuclear receptor isoform beta 2 [mice, embryonal carcinoma cell line, PCC7-MZ1, mRNA, 2971 nt]

Array Coordinate	Gene Family	Gene Name	Accession Number	Gene Description
O16	Signal Transduction	RIP	NM_009068	Mus musculus receptor (TNFRSF)-interacting serine-threonine kinase 1 (Ripk1), mRNA
B17	Signal Transduction	RxR-β	X66224	M.musculus mRNA for retinoid X receptor-beta (mRXR-beta)
C17	Signal Transduction	TANK	NM_011529	Mus musculus TRAF family member-associated Nf-kappa B activator (Tank), mRNA
D17	Signal Transduction	TRADD	AA013699	mh12f08.r1 Soares mouse placenta 4NbMP13.5 14.5 Mus musculus cDNA clone IMAGE:442311 5' similar to PIR:A56911 A56911 TRADD protein - human ;, mRNA sequence
E17	Signal Transduction	TRAF1	NM_009421	Mus musculus Tnf receptor-associated factor 1 (Traf1), mRNA
F17	Signal Transduction	TRAF2	L35303	Mus musculus TNF receptor associated factor 2 (TRAF2) mRNA, complete cds
G17	Signal Transduction	TRAF5	NM_011633	Mus musculus Tnf receptor-associated factor 5 (Traf5), mRNA
H17	Signal Transduction	TRAF6	NM_009424	Mus musculus Tnf receptor-associated factor 6 (Traf6), mRNA
I17	Signal Transduction	TRANK	NM_016764	Mus musculus peroxiredoxin 4 (Prdx4), mRNA
J17	Signal Transduction	TRIP	NM_011634	Mus musculus TRAF-interacting protein (Traip), mRNA
K17	Telomerase Related	TP1/Tep1	NM_009351	Mus musculus telomerase associated protein 1 (Tep1), mRNA
L17	Telomerase Related	TERT/TP2	NM_009354	Mus musculus telomerase reverse transcriptase (Tert), mRNA
M17	Telomerase Related	TR/TeRc	U33831	Mus musculus telomerase RNA component gene
N17	Telomerase Related	TRF1	NM_009352	Mus musculus telomeric repeat binding factor 1 (Terf1), mRNA
O17	Telomerase Related	TRF2	NM_009353	Mus musculus telomeric repeat binding factor 2 (Terf2), mRNA
B18	TNF Superfamily	DR-6	AW211328	uo79f09.y1 NCI_CGAP_Mam3 Mus musculus cDNA clone IMAGE:2648777 5' similar to TR:O75509 O75509 TNFR-RELATED DEATH RECEPTOR-6. ;, mRNA sequence
C18	TNF Superfamily	B-NGF	NM_013609	Mouse nerve growth factor (NGF) precursor mRNA, complete cds
D18	TNF Superfamily	NGF R	AF105292	Mus musculus nerve growth factor receptor mRNA, complete cds
E18	TNF Superfamily	TNF-α/TNFSF2	NM_013693	Mouse tumor necrosis factor (TNF) mRNA, complete cds
F18	TNF Superfamily	TNF-β/TNFSF1	NM_010735	Mus musculus lymphotoxin A (Lta), mRNA
G18	TNF Superfamily	OX40L/TNFSF4	NM_009452	Mus musculus tax-transcriptionally activated glycoprotein 1 ligand (Txgp1l), mRNA
H18	TNF Superfamily	FasL/TNFSF6	NM_010177	Mus musculus Fas antigen ligand (Fasl), mRNA
I18	TNF Superfamily	TRAIL/TNFSF10	NM_009425	Mus musculus tumor necrosis factor (ligand) superfamily, member 10 (Tnfsf10), mRNA
J18	TNF Superfamily	TRANCE/RANK L/TNFSF11	NM_011613	Mus musculus tumor necrosis factor (ligand) superfamily, member 11 (Tnfsf11), mRNA

Array Coordinate	Gene Family	Gene Name	Accession Number	Gene Description
K18	TNF Superfamily	TWEAK/TNFSF12	AF030100	Mus musculus TWEAK mRNA, partial cds
L18	TNF Superfamily	TALL-1/THANK/BAFF/TNFSF13B	AF119383	Mus musculus B-cell activating factor (Baff) mRNA, complete cds
M18	TNF Superfamily	LIGHT/TNFSF14	AB029155	Mus musculus mRNA for LIGHT protein, complete cds
N18	TNF Superfamily	TNF RI/TNFRSF1A	NM_011609	Mus musculus tumor necrosis factor receptor superfamily, member 1a (Tnfrsf1a), mRNA
O18	TNF Superfamily	TNF RII/TNFRSF1B	NM_011610	Mus musculus tumor necrosis factor receptor superfamily, member 1b (Tnfrsf1b), mRNA
B19	TNF Superfamily	OX40/TNFRSF4	NM_011659	Mus musculus tax-transcriptionally activated glycoprotein 1 (Txgp1), mRNA
C19	TNF Superfamily	CD40/TNFRSF5	NM_011611	Mus musculus tumor necrosis factor receptor superfamily, member 5 (Tnfrsf5), mRNA
D19	TNF Superfamily	Fas/TNFRSF6	NM_007987	Mus musculus Fas antigen (Fas), mRNA
E19	TNF Superfamily	RANK/TRANCE/TNFRSF11A	NM_009399	Mus musculus tumor necrosis factor receptor superfamily, member 11a (Tnfrsf11a), mRNA
F19	TNF Superfamily	OPG/TNFRSF11B	NM_008764	Mus musculus osteoprotegerin (Opg), mRNA
A24	Genomic DNA	GEN	N/A	Genomic DNA
B24	Housekeeping Genes	β2-Microglobulin	NM_009735	Mus musculus beta-2 microglobulin (B2m), mRNA
C24	Housekeeping Genes	β-Actin	X03672	Mouse cytoskeletal mRNA for beta-actin
D24	Housekeeping Genes	Cyclophilin A	NM_008907	Mus musculus peptidylprolyl isomerase A (Ppia), mRNA
E24	Housekeeping Genes	HPRT	J00423	Mouse hypoxanthine phosphoribosyltransferase (hprt) mRNA, complete cds
F24	Housekeeping Genes	L19	NM_009078	Mus musculus ribosomal protein L19 (Rpl19), mRNA
G24	Housekeeping Genes	Transferrin R	X57349	M.musculus mRNA for transferrin receptor
H24	Housekeeping Genes	α-Tubulin	M13446	Mouse alpha-tubulin isotype M-alpha-2 mRNA, complete cds
I24	Negative Control	1xTE Buffer	N/A	1x TE Buffer
J24	Negative Control	1xTE Buffer	N/A	1x TE Buffer
K24	Negative Control	pUC19	M77789	pUC19 cloning vector.
L24	Negative Control	E. coli b0658 gene	AE000170	*E. coli* b0658 gene (ybeX).
M24	Negative Control	E. coli b1444 gene	AE000241	*E. coli* b1444 gene, putative enzyme; not classified.
N24	Negative Control	E. coli b3535 gene	AE000430	*E. coli* b3535 gene (yhjR).
P24	Genomic DNA	GEN	N/A	Genomic DNA
