# Supplementary material for: Apoptosis gene profiling reveals spatio-temporal regulated expression of the p53/Mdm2 pathway during lens development
Source: Exp Eye Res. 2009 Jun 1;88(6):1137–51. doi: 10.1016/j.exer.2009.01.020 (PMC2706329; doi:10.1016/j.exer.2009.01.020)
Supplement: Supplementary file 9 [file mmc9.pdf]

Supplementary Table 2: Genes expressed at P7.

Gene ID	Gene Family	Mean Normalised Value
ABP1	Apoptosis-Related Factors	2.20
CAS/CSE1	Apoptosis-Related Factors	2.30
GSN	Apoptosis-Related Factors	14.86
ADAM17	Apoptosis-Related Factors	3.04
Ctsd	Apoptosis-Related Factors	13.55
HD	Apoptosis-Related Factors	3.26
API5	Apoptosis-Related Factors	2.97
Cytochrome p450 oxidoreductase	Apoptosis-Related Factors	3.38
HnrpA1	Apoptosis-Related Factors	21.71
SREBF2	Apoptosis-Related Factors	5.03
ATM	Apoptosis-Related Factors	2.41
DAD-1	Apoptosis-Related Factors	32.63
ICAD/DFFA	Apoptosis-Related Factors	4.30
PIN	Apoptosis-Related Factors	50.64
CAD	Apoptosis-Related Factors	2.70
Dap1	Apoptosis-Related Factors	8.10
Integrin-aV	Apoptosis-Related Factors	7.10
MFGE8	Apoptosis-Related Factors	2.75
Thrombospondin	Apoptosis-Related Factors	3.27
CD47	Apoptosis-Related Factors	2.40
DEDD	Apoptosis-Related Factors	4.02
CHML	Apoptosis-Related Factors	2.73
DNAse1	Apoptosis-Related Factors	2.35
REQ	Apoptosis-Related Factors	8.89
TIAL1	Apoptosis-Related Factors	15.97
CIDE-A	Apoptosis-Related Factors	2.43
TSSC3	Apoptosis-Related Factors	2.66
CLDN3	Apoptosis-Related Factors	2.04
Fem1B	Apoptosis-Related Factors	4.66
Mts-1	Apoptosis-Related Factors	23.74
TXN	Apoptosis-Related Factors	14.74
Cln3	Apoptosis-Related Factors	2.53
FLASH	Apoptosis-Related Factors	3.41
nNOS	Apoptosis-Related Factors	2.41
SAG-1	Apoptosis-Related Factors	4.26
Clu	Apoptosis-Related Factors	75.36
Galectin-3	Apoptosis-Related Factors	2.71
ODC	Apoptosis-Related Factors	17.27
GAPDH	Apoptosis-Related Factors	123.30
SARP-2/sFRP-1	Apoptosis-Related Factors	39.34
Cox-2/Ptgs2	Apoptosis-Related Factors	2.12
GPX1	Apoptosis-Related Factors	55.09
sFRP-5/SARP-3	Apoptosis-Related Factors	5.02
Caspase-7	Caspases and Regulators	18.92
Caspase-2	Caspases and Regulators	3.23
PARP	Caspases and Regulators	7.25
Cyclin G1	Cell Cycle Regulators	28.61
RBBP6/PACT/RBQ1	Cell Cycle Regulators	7.50

Gene ID	Gene Family	Mean Normalised Value
DP1	Cell Cycle Regulators	5.08
MDM2	Cell Cycle Regulators	5.15
APEX/Ref-1	Cell Cycle Regulators	11.18
Calcyclin	Cell Cycle Regulators	3.61
CBP	Cell Cycle Regulators	13.23
RBL2/p130	Cell Cycle Regulators	3.49
CDK2	Cell Cycle Regulators	2.66
CDK4	Cell Cycle Regulators	3.04
pRB	Cell Cycle Regulators	3.60
RBBP4/RbAp48	Cell Cycle Regulators	6.14
Cyclin D1	Cell Cycle Regulators	3.11
GM-CSF Ra	Cytokines and Receptors	13.28
Mannose 6-phosphate R	Cytokines and Receptors	5.02
M-CSF	Cytokines and Receptors	7.28
IGF-II	Cytokines and Receptors	4.29
TGF-b	Cytokines and Receptors	4.92
TGF-b2	Cytokines and Receptors	4.14
GAS1	Cytokines and Receptors	4.19
b-Actin	Housekeeping Genes	100.56
Cyclophilin A	Housekeeping Genes	47.11
L19	Housekeeping Genes	108.63
a-Tubulin	Housekeeping Genes	19.58
Bag-1	Mitochondrial Associated	11.44
Bcl-w	Mitochondrial Associated	12.40
Cytochrome C	Mitochondrial Associated	9.57
AKT/PKB	Signal Transduction	13.23
ASK1/MAP3K5	Signal Transduction	6.37
MEKK1	Signal Transduction	8.59
MYD118	Signal Transduction	28.61
DAP Kinase	Signal Transduction	4.18
E2F1	Signal Transduction	4.25
PKC-a	Signal Transduction	7.71
PTEN	Signal Transduction	8.97
GSK3B	Signal Transduction	10.24
14-3-3 eta	Signal Transduction	47.45
TP1/Tep1	Telomerase Related	13.26
NGF R	TNF Superfamily	15.33
