# Supplementary material for: Apoptosis gene profiling reveals spatio-temporal regulated expression of the p53/Mdm2 pathway during lens development
Source: Exp Eye Res. 2009 Jun 1;88(6):1137–51. doi: 10.1016/j.exer.2009.01.020 (PMC2706329; doi:10.1016/j.exer.2009.01.020)
Supplement: Supplementary file 10 [file mmc10.pdf]

Supplementary Table 3: Genes expressed at P14.

Gene ID	Gene Family	Mean Normalised Value
ABP1	Apoptosis-Related Factors	2.41
CAS/CSE1	Apoptosis-Related Factors	3.71
GSN	Apoptosis-Related Factors	15.89
PDCD1	Apoptosis-Related Factors	2.32
SIAH1	Apoptosis-Related Factors	3.36
ADAM17	Apoptosis-Related Factors	3.05
Ctsd	Apoptosis-Related Factors	15.06
HD	Apoptosis-Related Factors	21.96
PDCD2	Apoptosis-Related Factors	3.85
SREBF1	Apoptosis-Related Factors	5.43
API5	Apoptosis-Related Factors	3.90
Cytochrome p450 oxidoreductase	Apoptosis-Related Factors	4.62
HnrpA1	Apoptosis-Related Factors	13.35
PIGA	Apoptosis-Related Factors	2.26
SREBF2	Apoptosis-Related Factors	11.86
ATM	Apoptosis-Related Factors	2.20
DAD-1	Apoptosis-Related Factors	27.49
ICAD/DFFA	Apoptosis-Related Factors	15.03
PIN	Apoptosis-Related Factors	42.09
TDAG8	Apoptosis-Related Factors	5.02
CAD	Apoptosis-Related Factors	2.46
Dap1	Apoptosis-Related Factors	16.85
Integrin-αV	Apoptosis-Related Factors	14.61
PLA2G1B	Apoptosis-Related Factors	3.19
TFAR15	Apoptosis-Related Factors	4.00
CAV2	Apoptosis-Related Factors	2.37
DAXX	Apoptosis-Related Factors	4.32
MFGE8	Apoptosis-Related Factors	7.89
Thrombospondin	Apoptosis-Related Factors	6.04
CD47	Apoptosis-Related Factors	3.58
DEDD	Apoptosis-Related Factors	7.16
PRKR	Apoptosis-Related Factors	2.76
CHML	Apoptosis-Related Factors	2.84
DNAse1	Apoptosis-Related Factors	4.17
REQ	Apoptosis-Related Factors	10.51
TIAL1	Apoptosis-Related Factors	13.46
CIDE-A	Apoptosis-Related Factors	2.23
DNAse2	Apoptosis-Related Factors	2.43
CLDN3	Apoptosis-Related Factors	2.84
Fem1B	Apoptosis-Related Factors	5.91
Mts-1	Apoptosis-Related Factors	28.27
TXN	Apoptosis-Related Factors	12.88
Cln3	Apoptosis-Related Factors	2.90
FLASH	Apoptosis-Related Factors	3.02
SAG-1	Apoptosis-Related Factors	5.87
Clu	Apoptosis-Related Factors	63.25
Galectin-3	Apoptosis-Related Factors	6.67
ODC	Apoptosis-Related Factors	18.24
Cox-1/Ptgs1	Apoptosis-Related Factors	2.96
GAPDH	Apoptosis-Related Factors	169.87
P2RX1	Apoptosis-Related Factors	18.05
SARP-2/sFRP-1	Apoptosis-Related Factors	56.28
Cox-2/Ptgs2	Apoptosis-Related Factors	2.63

Gene ID	Gene Family	Mean Normalised Value
GPX1	Apoptosis-Related Factors	48.46
uPAR1	Apoptosis-Related Factors	2.47
sFRP-5/SARP-3	Apoptosis-Related Factors	6.87
Caspase-7	Caspases and Regulators	18.79
Sentrin/UBL1	Caspases and Regulators	3.57
Caspase-8	Caspases and Regulators	2.70
Survivin	Caspases and Regulators	2.76
Caspase-9	Caspases and Regulators	3.66
XIAP	Caspases and Regulators	4.35
FLIPL/Cash	Caspases and Regulators	3.25
Caspase-2	Caspases and Regulators	3.94
Caspase-3	Caspases and Regulators	4.87
PARP	Caspases and Regulators	7.69
PARP-2	Caspases and Regulators	3.62
Cyclin G1	Cell Cycle Regulators	51.28
RBBP6/PACT/RBQ1	Cell Cycle Regulators	6.50
DP1	Cell Cycle Regulators	6.76
Sp1	Cell Cycle Regulators	4.12
MDM2	Cell Cycle Regulators	16.80
TRP53/p53	Cell Cycle Regulators	7.01
53BP2	Cell Cycle Regulators	2.87
c-myc	Cell Cycle Regulators	3.23
APEX/Ref-1	Cell Cycle Regulators	10.20
p15INK4b/CDKN2B	Cell Cycle Regulators	2.63
Calcyclin	Cell Cycle Regulators	6.54
p19/NSG2	Cell Cycle Regulators	3.98
CBP	Cell Cycle Regulators	12.91
CDC2	Cell Cycle Regulators	3.81
CDK2	Cell Cycle Regulators	3.18
p300	Cell Cycle Regulators	4.01
CDK4	Cell Cycle Regulators	7.20
CDK5	Cell Cycle Regulators	4.63
pRB	Cell Cycle Regulators	3.62
RBBP4/RbAp48	Cell Cycle Regulators	7.75
Cyclin D1	Cell Cycle Regulators	5.61
RBP1	Cell Cycle Regulators	3.11
GM-CSF Ra	Cytokines and Receptors	15.58
Mannose 6-phosphate R	Cytokines and Receptors	5.36
IFN-g R1	Cytokines and Receptors	3.47
M-CSF	Cytokines and Receptors	8.33
AR	Cytokines and Receptors	3.43
IFN-g R2	Cytokines and Receptors	3.97
M-CSF R	Cytokines and Receptors	4.09
ART/Agrp	Cytokines and Receptors	3.00
IGF-I	Cytokines and Receptors	7.65
IL-4 Ra	Cytokines and Receptors	2.91
Prolactin	Cytokines and Receptors	2.55
Axl	Cytokines and Receptors	8.27
IGF-II	Cytokines and Receptors	3.55
TGF-β	Cytokines and Receptors	5.74
IGF R	Cytokines and Receptors	6.84
IL-10 Ra	Cytokines and Receptors	3.17
TGF-β2	Cytokines and Receptors	9.32

Gene ID	Gene Family	Mean Normalised Value
TGF-β3	Cytokines and Receptors	4.83
TGF-β RI	Cytokines and Receptors	3.08
GAS1	Cytokines and Receptors	5.05
β2-Microglobulin	Housekeeping Genes	2.91
β-Actin	Housekeeping Genes	105.54
Cyclophilin A	Housekeeping Genes	58.08
HPRT	Housekeeping Genes	5.03
L19	Housekeeping Genes	82.35
Transferrin R	Housekeeping Genes	3.69
α-Tubulin	Housekeeping Genes	32.61
Bag-1	Mitochondrial Associated	16.08
BAK	Mitochondrial Associated	5.24
Bax-a	Mitochondrial Associated	3.89
Bcl-2	Mitochondrial Associated	4.98
Bcl-w	Mitochondrial Associated	18.94
Bcl-x	Mitochondrial Associated	4.92
BID	Mitochondrial Associated	3.38
Cytochrome C	Mitochondrial Associated	7.13
Mcl-1	Mitochondrial Associated	8.04
A1	Mitochondrial Associated	6.53
AKT/PKB	Signal Transduction	9.73
RxR-b	Signal Transduction	3.75
TANK	Signal Transduction	4.76
ASK1/MAP3K5	Signal Transduction	9.33
MEKK1	Signal Transduction	9.92
Bcl-10	Signal Transduction	4.03
MYD118	Signal Transduction	20.37
NF-κB DNA binding subunit	Signal Transduction	4.22
TRAF2	Signal Transduction	3.42
CRADD	Signal Transduction	3.21
TRAF3/CRAF1	Signal Transduction	4.06
NF-kBp65	Signal Transduction	6.72
TRAF6	Signal Transduction	6.60
DAP Kinase	Signal Transduction	5.09
TRANK	Signal Transduction	4.52
PI-3 Kinase	Signal Transduction	2.88
TRIP	Signal Transduction	2.45
E2F1	Signal Transduction	7.25
PKC-a	Signal Transduction	9.20
FADD	Signal Transduction	3.44
FAN	Signal Transduction	3.98
PTEN	Signal Transduction	13.30
GSK3B	Signal Transduction	7.99
RARb2	Signal Transduction	4.72
14-3-3 eta	Signal Transduction	28.95
IKK-a	Signal Transduction	4.37
TP1/Tep1	Telomerase Related	7.85
TR/TeRc	Telomerase Related	5.80
NGF R	TNF Superfamily	12.18
FasL/TNFSF6	TNF Superfamily	3.93
TALL-1/THANK/BAFF/TNFSF13B	TNF Superfamily	4.60
